# Liquid Crystal Structure of Supercooled Liquid Gallium and Eutectic Gallium–Indium

**DOI:** 10.1002/adma.202104807

**Published:** 2021-08-01

**Authors:** Muhammad Yunusa, Alex Adaka, Amirreza Aghakhani, Hamed Shahsavan, Yubing Guo, Yunus Alapan, Antal Jákli, Metin Sitti

**Affiliations:** ^1^ Physical Intelligence Department Max Planck Institute for Intelligent Systems Heisenbergstrasse 3 70569 Stuttgart Germany; ^2^ Materials Science Graduate Program Advanced Materials and Liquid Crystal Institute Kent State University Kent OH 44242 USA; ^3^ Department of Physics Kent State University Kent OH 44242 USA; ^4^ Institute for Biomedical Engineering ETH Zurich Zurich 8092 Switzerland; ^5^ School of Medicine and College of Engineering Koç University Istanbul 34450 Turkey

**Keywords:** liquid crystals, liquid metals, metallic liquids, supercooled liquid gallium

## Abstract

Understanding the origin of structural ordering in supercooled liquid gallium (Ga) has been a great scientific quest in the past decades. Here, reflective polarized optical microscopy on Ga sandwiched between glasses treated with rubbed polymers reveals the onset of an anisotropic reflection at 120 °C that increases on cooling and persists down to room temperature or below. The polymer rubbing usually aligns the director of thermotropic liquid crystals (LCs) parallel to the rubbing direction. On the other hand, when Ga is sandwiched between substrates that align conventional LC molecules normal to the surface, the reflection is isotropic, but mechanical shear force induces anisotropic reflection that relaxes in seconds. Such alignment effects and shear‐induced realignment are typical to conventional thermotropic LCs and indicate a LC structure of liquid Ga. Specifically, Ga textures obtained by atomic force and scanning electron microscopy reveal the existence of a lamellar structure corresponding to a smectic LC phase, while the nanometer‐thin lamellar structure is transparent under transmission polarized optical microscopy. Such spatial molecular arrangements may be attributed to dimer molecular entities in the supercooled liquid Ga. The LC structure observation of electrically conductive liquid Ga can provide new opportunities in materials science and LC applications.

## Graphical abstract

The existence of a liquid crystal (LC) mesophase in metallic materials is counterintuitive. Typical LCs are made of covalently bonded organic molecules. This study reports on the observation of LC mesophases in supercooled liquid gallium and eutectic gallium–indium alloy. An LC texture of the lamellar structure similar to that of the smectic phase is unveiled at room temperature.

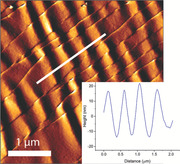

## 1 Introduction

Elemental gallium (Ga) has an unusual mixed covalent and metallic bond property at ambient pressure and temperature.^[^ ^1^ ^]^ Unlike any other simple liquid, supercooled liquid Ga is a complex liquid that exhibits both covalent and metallic character.^[^ ^2^ ^]^ The ability of elemental Ga to form an allotrope^[^ ^3^, ^4^, ^5^ ^]^ and its low‐melting temperature (29.8 °C) make it a promising candidate as a nontoxic metallic material with high thermal and electrical conductivity.^[^ ^6^ ^]^ In 1952, F.C. Frank hypothesized that an icosahedral short‐range order should be energetically favorable in supercooled liquids consisting of atoms of roughly spherical symmetry.^[^ ^7^, ^8^ ^]^ For decades, the anomalous structural ordering in supercooled liquid Ga has garnered significant attention in the scientific community. In previous attempts to describe the unusual property of liquid Ga, Tsay and Wang^[^ ^9^ ^]^ reported on the tetrahedron of Ga consisting of two dimers interlocked—having four indices with four‐atoms. One of the nearest neighbor atoms has a longer bond length compared to the rest of the neighbors, and therefore the tetrahedron is asymmetrical. In the case of the short‐lived covalent Ga dimers, a bond length of near 2.44 Å was attributed to the structural shoulder observed from the molecular dynamics simulations.^[^ ^2^ ^]^ However, a Ga–Ga pair separation of greater than 2.5 Å in a cluster structure is more likely to exist in liquid Ga.^[^ ^10^ ^]^ Thus, a medium‐range order of more than 20 Ga atoms is involved in the structural order in liquid Ga.^[^ ^11^ ^]^

To unravel the origin of the structural order in liquid Ga, classical molecular dynamics simulations have been conducted, which suggest the occurrence of particular clusters with some medium‐range order structure showing fourfold orientational symmetry in the liquid.^[^ ^2^, ^9^, ^10^, ^11^, ^12^ ^]^ Neutron and X‐ray scattering experiments revealed a scattering shoulder of structural order in liquid Ga.^[^ ^13^, ^14^, ^15^, ^16^ ^]^ Several interpretations were proposed for the structural order in liquid Ga based on the molecular dynamics simulations and X‐ray experiments, which include i) short‐lived dimeric bonding characteristic of the solid orthorhombic α‐Ga phase that persisted in the liquid state, and ii) structures beyond short‐range order—polyhedron clusters of four and five atom units.^[^ ^9^, ^11^, ^14^ ^]^ Recent studies highlighted that the dimeric bonding characteristics of α‐Ga phase do not persist in the liquid state, whereas an increasing fraction of fivefold symmetry and crystalline motifs exist in the liquid state.^[^ ^10^, ^12^, ^15^ ^]^ In addition, X‐ray analysis of liquid Ga droplet on diamond revealed the layering effect of Ga dimer (Ga_2_) molecules on a hard substrate.^[^ ^17^ ^]^ Recently, transmission electron microscopy analysis showed the thermally stable coexistence of liquid–solid phases in Ga and eutectic gallium indium (EGaIn) nanodroplets.^[^ ^18^, ^19^ ^]^ Both liquid Ga and EGaIn exhibit liquid–liquid phase transition behavior.^[^ ^4^, ^20^, ^21^, ^22^ ^]^ Hitherto, the long‐standing debate on the origin of the structural order in liquid Ga is still unsettled.

In this study, we demonstrate a possible liquid crystalline mesophase of supercooled liquid Ga sandwiched between various polymer‐coated glass substrates that induce homogeneous or homeotropic alignment in typical liquid crystal (LC) materials. The LC behavior is observed by the use of both reflective and transmission polarized optical microscopy (POM) techniques utilized in LC research, differential scanning calorimetry (DSC), atomic force microscopy (AFM), and scanning electron microscopy (SEM). The POM enabled the observation of anisotropic molecular alignment in the liquid metal. AFM study allows the characterization of lamellar structure in the liquid metal.

## 2 Results and Discussion

**Figure 1:**
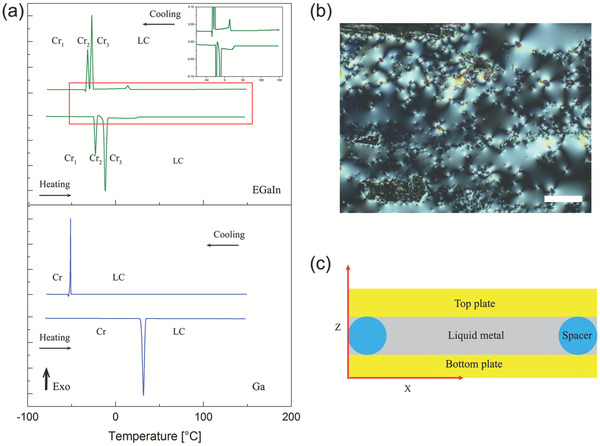
Thermal analysis and cell illustration of supercooled liquid gallium (Ga) sandwiched between two flat glass substrates. a) Differential scanning calorimetry (DSC) analysis results of Ga and EGaIn upon heating and cooling. The pseudo‐isotropic liquid structure that exhibits LC‐like property appears above the weakly first or second other phase transition temperature in EGaIn (19.6 °C) or the melting point of α‐Ga (30 °C) crystal during heating. The liquid recrystallized at different polymorph temperature of Ga, whereas in the case of pure Ga it crystallized at −51 °C indicating the stability of the supercooled state. b) R‐POM texture of the LC phase in supercooled Ga is similar to the Schlieren textures of nematic or smectic‐A (SmA) liquid crystals with homeotropic alignment. c) Cell geometry of confining liquid Ga or EGaIn. Scale bar: 200 µm.

DSC characterization of sandwiched supercooled liquid Ga shows first‐order transitions upon heating and cooling. On heating from −80 to 150 °C at 2 °C min^−1^, the pure Ga melts to pseudo‐isotropic liquid phase at around 30 °C, as shown in **Figure** **1**a. During cooling, the liquid Ga crystallizes to α‐Ga at −51 °C because of the supercooling effect of Ga (Table S1, Supporting Information). Although Ga is a polymorphic metal, only the peak that corresponds to the orthorhombic α‐Ga phase was detected in the DSC results at atmospheric pressure. Other polymorphs of Ga crystal are β‐Ga, γ‐Ga, and δ‐Ga, which are monoclinic, orthorhombic, and tetragonal, respectively.^[^ ^4^, ^5^ ^]^ They exist as metastable phases at atmospheric pressure at different temperatures. Polymorphism of Ga is a very well‐studied behavior due to size‐dependent and confinement effects. For instance, in a confined Ga particle, these four different phases of Ga structure are known to exhibit endothermic peaks during melting at 29.9 °C (α‐Ga), −16.2 °C (β‐Ga), −19.4 °C (δ‐Ga), and −35.6 °C (γ‐Ga), respectively.^[^ ^4^, ^5^ ^]^ Interestingly, these polymorphs could be detected in the DSC curve of the EGaIn alloy, which is a low melting point eutectic liquid (Figure 1a and Table S1, Supporting Information).^[^ ^23^, ^24^ ^]^ Chen et al.^[^ ^25^ ^]^ reported on the metastable phases in gallium–indium alloy and attributed the endothermic peaks, which are at −11 and −22 °C to a metastable solid‐solution composed of β‐Ga and δ‐Ga. We observed similar peaks at −11.87 and −23 °C, which are very close to their results (Figure 1a). Indeed, the metastable phases behave like solid solution where they segregate from the alloy during cooling from the solid–liquid solution until the last phase is formed. The formation of such phases is size dependent and is unusual. In a recent study, phase segregation has been shown during interfacial solidification of eutectic bismuth‐gallium alloy with a melting point slightly above room temperature (RT).^[^ ^26^ ^]^ However, in another study using a similar approach, interfacial phase‐separation driven pattern formation during the crystallization of elemental Ga was not observable.^[^ ^27^ ^]^ The transition from liquid to the solid phase is a first‐order transition with an enthalpy of 136.91 J g^−1^ (Table S1, Supporting Information). We performed DSC experiments at higher temperatures up to 400 °C, which did not reveal any first‐order phase transition peak in either Ga or EGaIn.

In a thin (4 to 6 µm) layer of the supercooled liquid Ga prepared on a clean glass substrate at RT with open‐air boundary, a grayish texture is observed using a circularly polarized light in reflective polarized optical microscopy (R‐POM) (Figure 1b). The fact that the reflected light is not extinguished between the crossed polarizers means anisotropic reflection at the Ga‐substrate interface. The observed texture resembles the Schlieren textures of various LC phases subjected to a homeotropic boundary condition (boundary layer tends to align the LC director normal to the substrate).^[^ ^28^ ^]^ There are only two brush defects seen, which means the presence of only 1/2‐strength defects. In LCs, such textures may be due to pretilt of the elongated building blocks, or may indicate a biaxial nematic or smectic phase.^[^ ^29^, ^30^ ^]^ Note that the open‐air surface of the liquid maybe covered with a Ga oxide layer with about 3‐nm thickness. The highly transparent oxide layer occurs naturally when liquid Ga or EGaIn is exposed to open ambient air.^[^ ^31^, ^32^ ^]^ This may cause some pretilt at the air–Ga interface, but otherwise the optical property of thin liquid Ga is unaffected due to the optical transparency of the oxide layer.^[^ ^31^, ^33^ ^]^ The contact angle measurement of liquid Ga on clean glass surface at ambient temperature shows smaller wetting angle compared to other surfaces (Figure S1, Supporting Information). To investigate the effect of the amorphous gallium oxide layer on the liquid Ga film in open air, crystallization study of bulk liquid Ga was carried out under R‐POM (Figure S2, Supporting Information). During crystallization of the bulk liquid at RT, solid orthorhombic crystal of Ga phase was obtained from the initial liquid phase, which was covered with the nanoscale amorphous gallium oxide layer. It is important to know that the crystallized Ga exhibits an optical anisotropy, which can be attributed to the RT orthorhombic Ga crystal phase. The surface oxide layer has no influence on the reported optical behavior of the liquid metals. For instance, Zavabeti et al. reported on the exfoliation of the amorphous gallium oxide on liquid metal droplet.^[^ ^34^ ^]^ During the first order crystallization of Ga, a large area of highly oriented crystalline Ga film was observed under R‐POM from the liquid phase sandwiched between clean glasses (Figure S3 and Movie S1, Supporting Information). Furthermore, powder X‐ray diffraction (PXRD) investigation on the surface of bulk Ga crystallized at RT in open air reveals the diffraction peak of α‐Ga (Figure S4, Supporting Information). Energy‐dispersive X‐ray spectroscopy (EDS) of Ga crystal obtained at RT confirms the presence of gallium oxide on the surface (Figure S5, Supporting Information).

**Figure 2:**
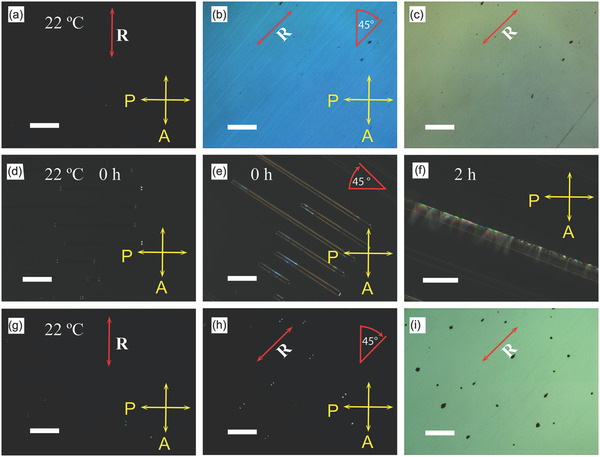
Reflection polarized optical microscope (R‐POM) images of the liquid Ga cell and EGaIn alloy in a microchannel. a–c) R‐POM images of a planar texture taken at 22 °C with crossed polarized (a), 45° sample rotation (b), and without analyzer (c). Transmission POM images of microchannels filled with EGaIn alloy (15 µm width, 55 µm period, and 17 µm depth). d–f) Microchannel at 0 h after preparation with crossed polarized (d), crossed polarized rotated 45° (e), and crossed polarized rotated 45° after 2 h depicting parabolic focal conic domains (f). g–i) R‐POM images of the homeotropic texture with crossed polarizers (g), rotated 45° (h), and without analyzer (i). The red arrows represent the flow directions. The thickness of planar and homeotropic cells are 40 and 17 µm, respectively. Scale bars: 100 µm (a–e), 50 µm (f), and 200 µm (g–i).

R‐POM observations in the bulk supercooled liquid Ga were carried out in cells where the material was sandwiched between two glass plates coated with a polymer layer that provides either planar (homogeneous) or homeotropic alignment for LCs (Figure 1c). For instance, a planar alignment where the director is parallel to the substrates was achieved by confining the liquid in between two unidirectionally rubbed poly(methyl methacrylate) (PMMA) plates separated by a gap ranging from 5 to 50 µm at 22 °C. In **Figure** **2**, the planar condition of the supercooled liquid Ga in the PMMA cell with 40‐µm separation is illustrated. A crossed‐polarized optical image of the cell appeared dark when one of the polarizers was parallel to the flow direction of the liquid between the plates during preparation (Figure 2a). When the director or the optical long axis was aligned parallel to one of the polarizers, the sample was extinguished. However, when we rotated the sample between the crossed polarizers, the texture brightens, reaching the brightest state when the rubbing direction is at 45° with respect to the polarizers (Figure 2b). A POM image without an analyzer is shown in (Figure 2c). The red arrow indicates the direction of the rubbing (R). The PMMA surface shows a strong planar anchoring of the liquid Ga clusters.

POM textures were also studied in sandwich cells at RT, where one of the substrates contained polydimethylsiloxane (PDMS) microchannels (15 µm width, 55 µm period, and 17 µm depth) (Figure 2d,e). The channels infiltrated with EGaIn liquid at 22 °C immediately show planar alignment. After 2 h, the parabolic focal conics (PFC) type domains^[^ ^35^ ^]^ appeared in the channels with the long axis of the elongated parabolas pointing perpendicular to the channel walls (Figure 2f). Note that the images in Figure 2d–f were obtained in the transmission POM (T‐POM). Light absorption spectra of Ga and EGaIn at certain wavelengths were measured using UV/vis/NR (Figure S6, Supporting Information). Such kind of PFC defects are well known in LCs with either smectic layers in smectic LCs or pseudo‐layered structures of chiral nematic^[^ ^36^ ^]^ or the twist‐bend nematic phases.^[^ ^30^ ^]^ PFC appears when either the layer spacing decreases, for example, on cooling or when the films are dilated.^[^ ^37^ ^]^ These observations indicate the presence of some kind of true or pseudo‐layer structure of the supercooled liquid Ga. The direction of the PFC domains also indicates that the layers are parallel to the channel wall. This is due to the shear alignment that allows flow only along the channel wall and the smectic layers.^[^ ^38^ ^]^ The lamellar structure behavior of Ga was further investigated by flow‐assisted dilation of the liquid film (see Figure S7, Supporting Information). To dilate the smectic‐type layers, both liquid Ga and EGaIn were squeezed from a cavity between clean glass plates where one of the glass surfaces was coated with 50‐µm thick acrylic adhesive tape (optically clear double‐sided adhesive, Thorlabs, Inc.). As a result, the lamellar organization was obvious where PFC are clearly visible under R‐POM at RT with layers parallel to the surface (Figure S8, Supporting Information).^[^ ^37^ ^]^ In a surface stabilized horizontal domain texture of liquid Ga and EGaIn, typically nanometer‐thick layer, POM observation using transmission light illumination was conducted showing optical anisotropy (Figures S9 and S10, Supporting Information).

The PXRD measurement using Cu‐kα1 shows a downward shift of sharp peaks during heating above 70 °C in a thin glass capillary (Figure S11, Supporting Information). The diffraction patterns cannot be indexed by any known gallium oxide or gallium hydroxide phase structure. Thus, the diffraction result could be attributed to a fluidic smectic behavior of the Ga clusters in the supercooled liquid with layer arrangement. Note that the X‐ray was conducted in unaligned thin glass capillaries. The peak positions are less reproducible (much wider) and the positional symmetry is poor (Figure S12, Supporting Information). Additionally, we conducted the X‐ray diffraction on liquid capillary (300‐µm diameter) and a glassy thin film using Ag radiation at RT to further examine the structure (Figure S13, Supporting Information). Although no sharp X‐ray scattering was observed in the liquid and glassy film using Ag‐kα1 radiation, the structure of the liquid and the film resembles that of amorphous or supercooled liquid Ga with a shoulder on the main structural peak.^[^ ^14^, ^16^, ^39^, ^40^ ^]^ The scattering features may be explained by the dense packing of short‐range molecules, clusters, or tetrahedral structures. A similar scattering pattern has been reported in the LC of graphene oxide.^[^ ^41^ ^]^ Although the X‐ray diffraction measurement is inconclusive, it nevertheless sheds light on the structure of the liquid metal. Determination of the actual cluster size in an aligned configuration using a high‐energy X‐ray is a future work.

**Figure 3:**
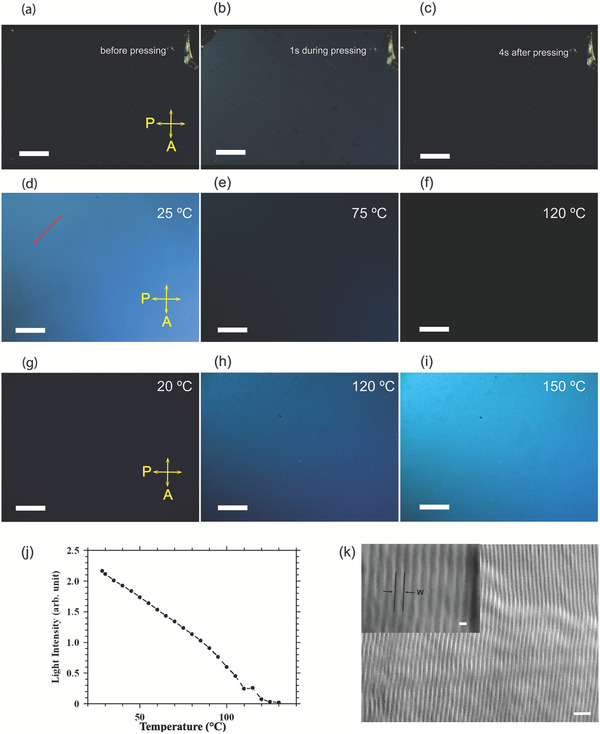
Thermotropic LC structure behavior of the supercooled liquid Ga. Illustration of the pressing‐induced realignment effect. a,c) The R‐POM image is dark before pressing (a) and 4 s after pressing (c) indicating a homeotropic alignment. b) During pressing, the texture is bright showing a flow‐induced realignment. d–f) Temperature dependence of the anisotropic LC structure in homogeneous cell revealed in R‐POM with 50 µm thickness. The brightness diminished at high temperature because of realignment of the molecular optical long axis with the polarizers. f) When the homogeneous cell was rotated, a brightness change was observed. g) Homeotropic cell of polyimide treated ITO glass (17 µm thickness) during cooling after transition from M1 to M2. h,i) In the M1 phase above 120 °C, the homeotropic texture is comparable to the homogeneous texture in (d), which indicates degenerate planar alignment. j) Reflectance intensity measurement of the planar geometry with respect to temperature change. At 120 °C, the intensity decreased almost to zero. k) Scanning electron microscope image of the solidified Ga demonstrating stripe texture from the bulk film, where *w* represents the width of the stripe of ≈40 nm. Scale bars: 400 µm (a–c), 100 µm (d–f), 200 µm (g–i). k) 200 and 40 nm (inset).

In a 17‐µm‐thick Ga‐filled cell with substrates treated by a polyimide that usually promotes homeotropic alignment (director perpendicular to the substrate) for thermotropic LCs, the texture appeared dark in R‐POM with crossed polarizers even when the sample was rotated 45° with respect to the polarizers (Figure 2h,i). Homeotropic textures are usually observed for the nematic and smectic‐A (SmA) LC phases between homeotropic anchoring surfaces, where the material appears pseudo isotropic with the optical axis normal to the substrates. The mechanical shear force exerted by pressure on the substrate with a tweezer induces an anisotropic reflection that relaxes in seconds due to reversible reorientation of the director (**Figure** **3**a–c and Movie S2, Supporting Information). Such alignment effects and the shear‐induced realignment are typical to conventional thermotropic LCs and strongly indicate the LC structure of liquid Ga. Additionally, focal conic domain has been observed on the Ga droplet (Figure S14, Supporting Information) akin to cholesteric or smectic droplet with onion‐like structures.^[^ ^42^, ^43^ ^]^ In a homeotropic treated cell (50 µm thickness), the texture is nearly uniform and appeared dark between crossed polarized light under R‐POM. When uncrossed one of the polarizers, the texture splits into brighter and darker bands that exchange the brightness with opposite uncrossing directions (±6°). This indicates the existence of some domains with opposite optical activity (see Figure S15, Supporting Information). Similarly, when liquid Ga is slightly sheared between clean glass plates with a cell gap of 10 µm, large area domains are split into brighter and dark bands following the uncrossing of the polarizers in different directions. This is indicative of domains with the opposite optical activities (see Figure S16, Supporting Information).

In order to investigate the thermotropic LC property of the structure in liquid Ga, a planar cell with a 50‐µm thickness was subjected to heating/cooling at 2 °C min^−1^ (Figure 3d). The optical texture was monitored with R‐POM and the high‐temperature phases were studied. Several observations were noted while heating the cell starting from the RT mesophase (M2). The long axis of the Ga nanoclusters and the rubbing direction were oriented at 45° with respect to the polarizers. In such position, the intensity of the cell gradually decreased with the increasing temperature (Figure 3e). At temperatures above 120 °C, the cell became completely dark (Figure 3f). Interestingly, the planar cell appeared bright again when rotated 45° from the initial orientation. We refer to the mesophase above 120 °C as M1. By further heating, birefringent bubble domains started to appear up to 145 °C. The texture of M2 in polyimide treated ITO cell (17 µm) was dark when cooled from the M1 phase (Figure 3g). The texture of M1 in the homeotropic cell at 120 and 150 °C exhibits uniform birefringence that can be attributed to SmC phase (Figure 3h,i).^[^ ^44^ ^]^ There was no front propagation during phase transition from M1 to M2, which indicates a second‐order transition usually found in SmA–SmC. In a planar cell with a 10‐µm film thickness, the intensity of the anisotropic reflection decreases to almost zero at 120 °C (Figure 3j).

Ga film (300‐µm thick) obtained by slow cooling between PDMS and clean glass substrates shows stripe domains under SEM imaging. The film was fractured after plunging into liquid nitrogen. The SEM image was taken from the bulk inner region of the fractured bulk solid Ga film, and the film was kept at −20 °C throughout the imaging process (Figure 3k). The width (*w*) of the stripes is 40 nm according to the SEM observation. The lamellar steps within the bulk material exhibit a well‐defined layering of Ga (Figure S17, Supporting Information). In a slowly cooled (1 °C min^−1^) capillary filled with liquid Ga, highly oriented stripes are observed in the bulk structure with a thickness of roughly 40 nm (Figure S18, Supporting Information). However, when quenched the capillary (500 µm in diameter) with Ga in liquid nitrogen, solid structures of different lamellar orientations emerged according to our SEM observation (Figure S19, Supporting Information).

**Figure 4:**
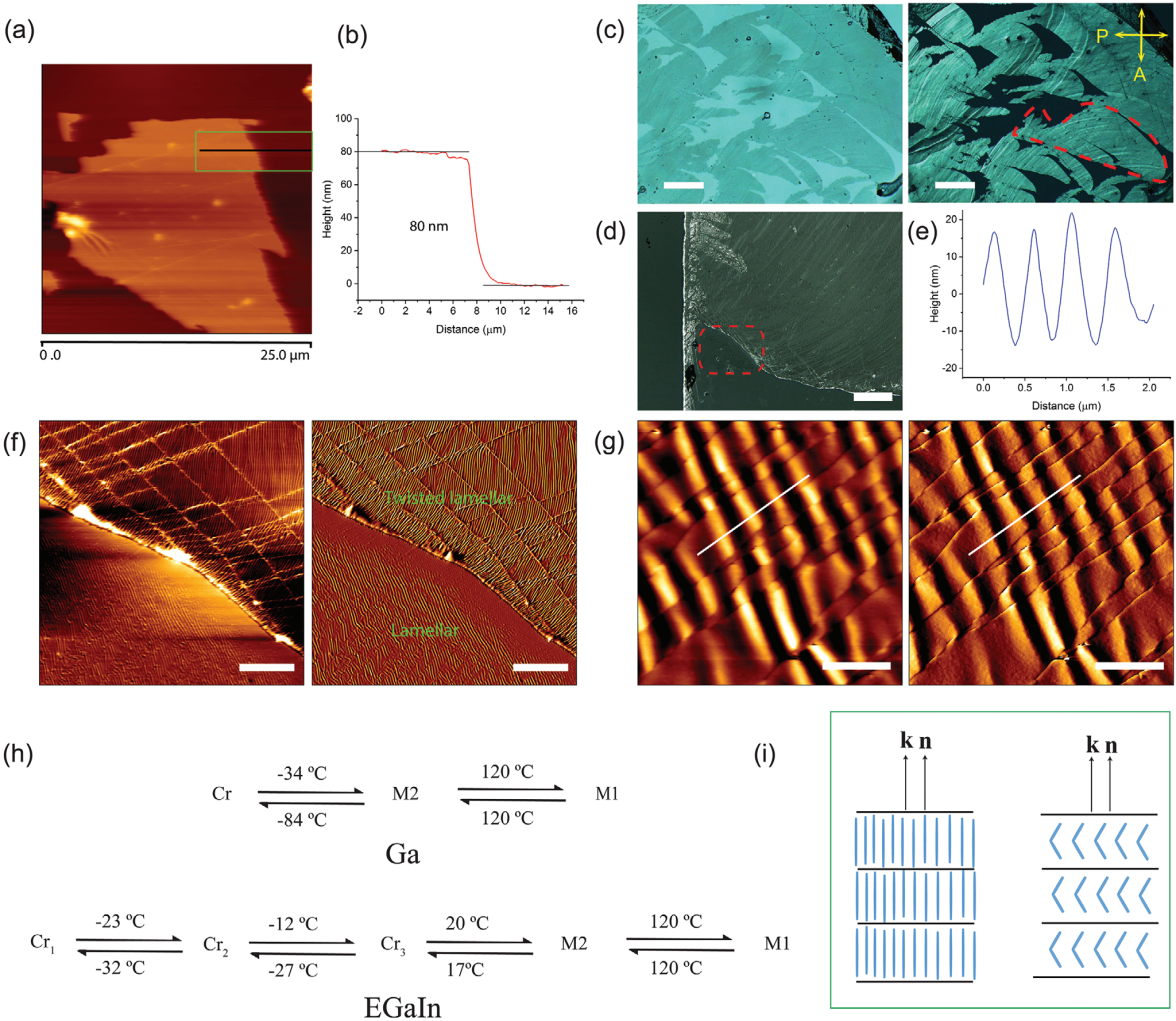
Atomic force microscopy (AFM) topography images of the lamellar region. a) AFM height image of the exfoliated lamellar on the PDMS surface. b) Height measurement of green area in (a) showing thickness of 80 nm consisting of double lamellar. c) Optical image of a transparent exfoliated lamellar. Bright field image (left) and crossed‐polarized transmission POM (right). d) Optical image of large area exfoliated lamellar layer structure. e) Cross‐sectional analysis of layer undulation in the selected area in (g). f) Height image (left) and vertical deflection topography (right) of lamellar structure. The imaged was obtained from the red dashed area in (d). g) Height image (left) and vertical deflection topography (right) of lamellar undulation in the bottom region of (f). h) Phase sequence according to R‐POM observations. i) Illustration of the proposed molecular orientation of the room temperature M2 phase. Scale bars: 100 µm (c), 100 µm (d), 10 µm (f), and 1 µm (g).

The characterization of the lamellar structure via AFM was employed to measure the layer thickness of the nanometer‐thick transparent films. We have exfoliated transparent lamellar films of tens of nanometer by the use of PDMS and clean glass surfaces at RT. Before the liquid‐phase exfoliation, the liquid metal was sandwiched between the two surfaces as shown in the schematic in Figure S20, Supporting Information. In this orientation, the PDMS surface enabled strong planar anchoring of both liquid Ga and EGaIn (Figure S21, Supporting Information). Mechanical exfoliation (repeated peeling) of layered materials has been used to obtain monolayers of graphene sheets with a few nanometer thickness from a highly oriented pyrolytic graphite.^[^ ^45^ ^]^ The liquid‐phase exfoliated lamellar layers are transparent with thickness up to 120 nm, and are parallel to the PDMS surface (Figure S22, Supporting Information). **Figure** **4**a illustrates the height thickness of a transparent lamellar of bilayer structure (80 nm) at RT. The thickness in Figure 4b corresponds to the line analysis in Figure 4a. The layers transferred onto the PDMS (≈1 mm thick) surface after 2 h of cell preparation clearly show the planar alignment of the lamellar structures. In a previous study, Kochat et al. exfoliated a few nanometer atomically thin Ga sheet through solid‐melt exfoliation on various surfaces.^[^ ^46^ ^]^ In contrast to their approach, the thickness of a lamellar in this study is in the order of tens of nanometer (40 nm) (Figure S23, Supporting Information). The PDMS surface was characterized as a control. The T‐POM images of a monolayer lamellar are shown in bright field and crossed‐polarized POM (Figure 4c). The red dashed area shows a demarcation of a large area lamellar structure. The lamellar thickness measured using AFM is consistent with the thickness of the stripes measured in SEM (Figure 3k). Figure 4d represents a large area of optical micrograph of spatially organized lamellar sheets with layer undulation of either smectic or columnar LC phase.^[^ ^47^ ^]^ When stacked lamellar are twisted on each other, a pattern similar to Moiré superlattice was visualized.^[^ ^48^ ^]^ The AFM micrographs in Figure 4f shows the height image (left) and vertical deflection (right) of measured topography (50 × 50 µm^2^ area), respectively. A small area (4 × 4 µm^2^) scan of a lamellar with undulation is depicted in Figure 4g with the height image on the left and the vertical defection on the right. The period of undulation is 500 nm with an amplitude of 15 nm as seen in the line analysis (Figure 4e). The proposed phase sequence of the LC phase of Ga obtained from the R‐POM observation is illustrated in Figure 4h. There was no detectable enthalpy in the DSC measurement corresponding to the second order transition at 120 °C in both Ga and EGaIn. The possible molecular orientation of the room temperature M2 phase is depicted in Figure 4i. It is important to mention that the structure of the supercooled liquid Ga sandwiched between differently treated surfaces used in conventional LC study is very stable at RT and below (Figure S24, Supporting Information). Liquid Ga can be overcooled down to −84 °C without crystallization. However, EGaIn crystallizes into different polymorphs upon cooling as dictated by the DSC results in agreement with the POM observation.

**Figure 5:**
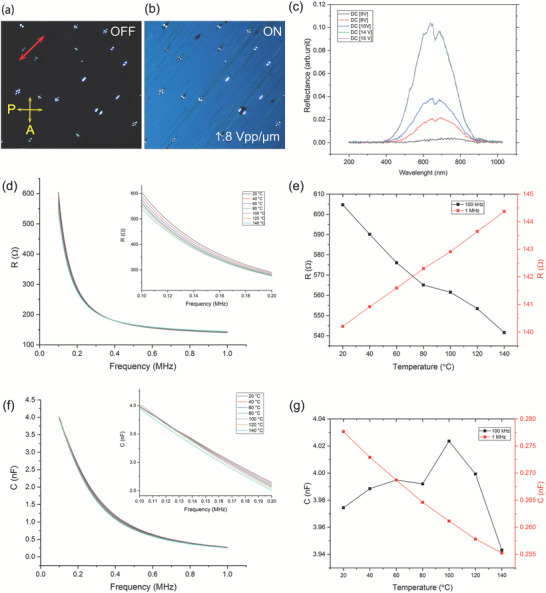
Electro–optics and electrical characterization of the LC structure of supercooled liquid Ga. a,b) Electro–optics switching of a Ga film at RT with DC electric field in a 10‐µm polyimide (PI) treated cell. c) Applied DC voltage dependence of the reflection intensity. The reflection intensity covers a broadband wavelength from 400 to 900 nm. d) Temperature‐dependent resistance behavior of Ga measured across a homeotropic cell consisting of an ITO‐treated glass with 17 µm thickness. The resistance across the cell depends on the frequency. e) The resistance was measured at two different frequencies: 100 kHz and 1 MHz. At low frequencies (100–400 kHz), the resistance decreases with temperature, while at higher frequencies (1 MHz) the resistance increased with temperature. f,g) Temperature‐dependence behavior of the capacitance across the same cell at different frequencies. Both resistance and capacitance were measured during cooling from 140 °C.

Counterintuitively, the molecular behavior of LC‐Ga enabled electro–optics switching, where an anisotropically reflecting state was induced via the application of 1.8 V µm^−1^ electric field at RT in between two ITO‐coated glasses separated by 10 µm, as shown in **Figure** **5**a,b and Movie S3, Supporting Information. The homeotropic ITO cell (Figure S25, Supporting Information) was prepared by a polyimide coating in which the electro–optical switching was realized by applying a square wave alternating field (AC) of 1.9 V µm^−1^ at 0.5 and 1 Hz across the cell (Movies S4 and S5, Supporting Information). The reflected light intensity increased with the strength of the applied field, as can be seen in Figure 5c.

Owing to the high electrical conductivity of the supercooled liquid Ga, the applied DC field across the cell generated heat in the liquid via Joule heating and thus the temperature of the cell rose to around 50 °C. The temperature within the cell was uniform due to the high thermal conductivity of liquid Ga. During this localized heating, there is less thermal dissipation through the bulk glass plates. The electro–optic effect was found to be independent of the temperature until 130 °C, where the electric field dependence of the anisotropic reflection vanished. The underlying physical mechanism of this electro–optical phenomenon is not quite clear yet and will be the subject of future studies. The temperature dependence of resistance across the cell was characterized in a 17‐µm thick ITO coated cell. The resistance of the liquid Ga cell shows frequency‐dependent behavior (Figure 5d). At low frequencies (100–400 kHz), the resistance increased linearly with temperature during cooling from 140 to 20 °C. On the contrary, the resistance decreases with decreasing temperature at a higher frequency. In Figure 5e, the resistance is depicted for 100 kHz and 1 MHz frequencies. Further characterization of capacitance across the homeotropic Ga cell shows temperature dependence at higher frequencies. Figure 5f shows the capacitance‐temperature effect of the cell. The capacitance linearly correlates with the temperature at higher frequencies (1 MHz). The capacitance measurements at frequencies of 100 kHz and 1 MHz are plotted in Figure 5g. The electrical current passing across the cell increased with the applied voltage due to the high electrical conductivity of the liquid Ga (Figure S26, Supporting Information). The current passing through the cell appeared to be proportional to the applied DC voltage.

When heated to high temperatures above 130 °C, periodic strands of nanocrystals nucleate around the birefringence bubbles during isotropic transition at around 145 °C in both the Ga and EGaIn liquids (Figure S27a,b, Supporting Information). The R‐POM images were taken at 22 °C after slow cooling to RT at 2 °C min^−1^. SEM images taken from the quenched planar‐aligned cell of EGaIn show submicron‐size needles with a periodicity of about 100 nm (Figure S27c,d, Supporting Information). The images clearly show the spatial arrangement of the helical strands or needle‐like domains with a half‐pitch that ranges from hundreds of nanometers to 2.5 µm. Near to a birefringence bubble in a homeotropic ITO cell, well‐defined layered structures of Ga were visualized with vertical layer orientation (Figure S28, Supporting Information). To prepare the glassy Ga samples, the samples were plunged into liquid nitrogen before the SEM observation. The samples were kept at −20 °C during imaging to prevent any structural reorganization and damage by the electron beam during SEM characterization. It is worth mentioning that a glass transition of Ga metal was reported previously in a slowly quenched Ga film, which reassured the molecular behavior of the liquid phase.^[^ ^27^ ^]^

## 3 Conclusion

To summarize, in this work, we unraveled the LC structure in the supercooled liquid Ga and EGaIn. This could help explain the unusual cluster formations in liquid Ga and may pave the way for the development of new physics to explain the anomalous behavior of metallic liquids and open a new avenue for LC‐related potential applications of metallic liquids. This work has potential impact in future low dimensional electronic devices.

## 4 Experimental Section

### Materials

Commercially available pure liquid Ga (99.99999%, Sigma Aldrich) and EGaIn (Ga 75.5%–In 24.5%, 99.99% Sigma Aldrich) were purchased. Thermal annealing was performed to induce the supercooling.

### Microscopic Characterization

A SEM (Zeiss Ultra 500 Gemini SEM, Carl Zeiss Inc., Oberkochen, Germany) was used to characterize the microstructure and morphology of Ga films. The fractured films were examined in SEM at −20 °C to prevent structural damage. The sample was kept at −20 °C throughout the imaging process using built‐in cooling system (Figure S29, Supporting Information).

AFM of transparent thin films of lamellar was performed using Bruker JPK Nanowizard 4 in contact mode. The authors used an Arrow‐Contr‐10 cantilever calibrated at 14 kHz resonance frequency and 0.2 N m^−1^ force constant.

X‐ray diffraction patterns were obtained using Stoe stadi‐P with Cu‐kα1 (1.54 Å) and Ag‐kα1 (0.56 Å) in transmission using a goniometer for vertically mounted capillaries (Borosilicate capillary, WJM‐Glas Müller GmbH). A flat thin‐film diffraction in transmission was obtained by sandwiching 50 µm Ga film between two Kapton films. The film was slowly cooled at 1 °C min^−1^ to up to −125 °C to obtain the stabilized glassy film.

### Differential Scanning Calorimetry Analysis

Thermal analysis (with modulation at 0.32°) was obtained by Discovery DSC 2500 (TA Instrument) using copper, alodined Al, and graphite using inert nitrogen purge. All pan materials showed consistency in the thermal peak determination whether in open or closed pan.

### Polarized Optical Microscope Imaging

Polarized light microscope (ZEISS Axio Imager.Z2, Carl Zeiss Inc., Oberkochen, Germany) equipped with polarizers and rotating stage was used for optical observations in reflection mode to determine the phase sequence of the LC phase. Linkam (THMS600) temperature‐controlled stage mounted on ZEISS Axio Imager.Z2 enabled in situ thermal control of the sample and subsequent imaging during experiments. Precise cooling of the sample was achieved via liquid nitrogen cooling through the LNP96 cooling option.

### Light Spectroscopy Technique

Light absorption spectrum of samples was obtained using Perkin Elmer UV–vis–NR, lambda 1050+. Compact CCD spectrometer (Thorlabs) for wavelengths ranging from 200 to 1000 nm was used to record reflectance intensity.

### Electro–Optics, Resistance, and Capacitance Characterization

DC voltage (DC power supply, KEYSIGTH U80001A) of 18 V was applied across 10 µm‐thick films. For AC actuation, a function generator (Tektronix AFG3102C) was combined with a high‐frequency power amplifier (TREK MODEL 2100HF) to deliver 36 Vpp (peak‐to‐peak voltage) using square or sine wave function. A mixed channel oscilloscope (Tektronix) device monitored the frequency response of the actuation signal. Resistance and capacitance characterization were performed using KEYSIGTH ENA Network Analyzer (5 Hz–3 GHz). An empty ITO cell was used for calibration before the measurement.

## Conflict of Interest

The authors declare no conflict of interest.

## Supporting information

Supporting information

Supplemental Movie 1

Supplemental Movie 2

Supplemental Movie 3

Supplemental Movie 4

Supplemental Movie 5

## Data Availability

The data that supports the findings of this study are available in the supplementary material of this article.
