# Supplementary material for: Liquid Crystal Structure of Supercooled Liquid Gallium and Eutectic Gallium–Indium
Source: Adv Mater. 2021 Aug 1;33(38):2104807. doi: 10.1002/adma.202104807 (PMC11468993; doi:10.1002/adma.202104807)
Supplement: Supplementary file 1 — Supporting information [file ADMA-33-2104807-s006.pdf]

© 2021 Wiley-VCH GmbH

ADVANCED MATERIALS

Supporting Information

for *Adv. Mater.,* DOI: 10.1002/adma.202104807

Liquid Crystal Structure of Supercooled Liquid Gallium and Eutectic Gallium–Indium

Muhammad Yunusa, Alex Adaka, Amirreza Aghakhani, Hamed Shahsavan, Yubing Guo, Yunus Alapan, Antal Jákli, and Metin Sitti**

Supplementary Materials

Liquid Crystal Structure of Supercooled Liquid Gallium and Eutectic Gallium-Indium

Muhammad Yunusa, Alex Adaka, Amirreza Aghakhani, Hamed Shahsavan, Yubing Guo, Yunus Alapan, Antal Jákli, and Metin Sitti

Table S1. Phase transition enthalpies taken from DSC analysis at 2 °C/min heating and cooling rate.

Material	Phase transition	T [°C] (ΔH [Jg^{-1}]) Heating cycle	T [°C] (ΔH [Jg^{-1}]) Cooling cycle
Ga	Cr—LC	+31.71 (79.78)	-51.21 (136.91)
EGaIn	Cr_1—Cr_2	-23.07 (12.22)	-32.17 (12.72)
	Cr_2—Cr_3	-11.87 (37.47)	-27.16 (25.76)
	Cr_3—LC	+19.61 (3.11)	+17.67 (1.70)

Cr: crystal phase. Subscript 1, 2, and 3 represent different polymorphs of Ga.

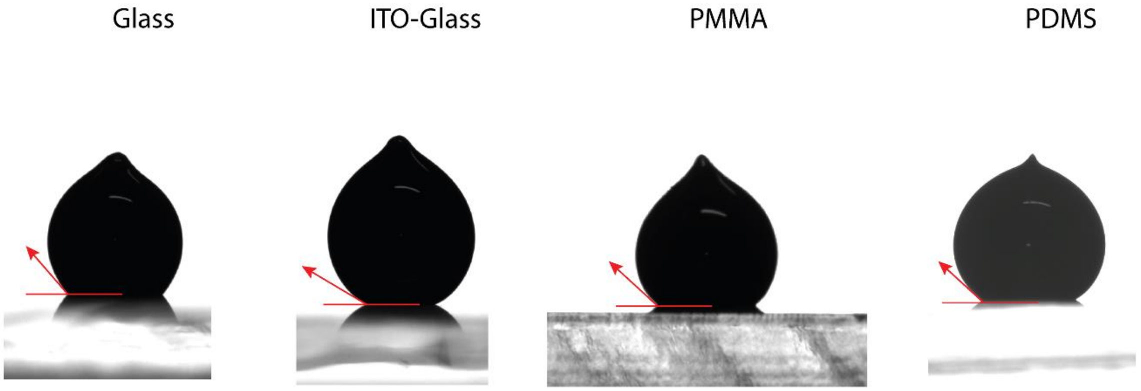

Substrate	Contact angle (º)
Glass	129.5
ITO-Glass	153.6
PMMA	140.2
PDMS	140.5

Figure S1. Contact angle analysis of the supercooled liquid Ga on various material surfaces. Note that, the surface of the droplets is covered with the native oxide layer as the measurements were performed at ambient conditions.

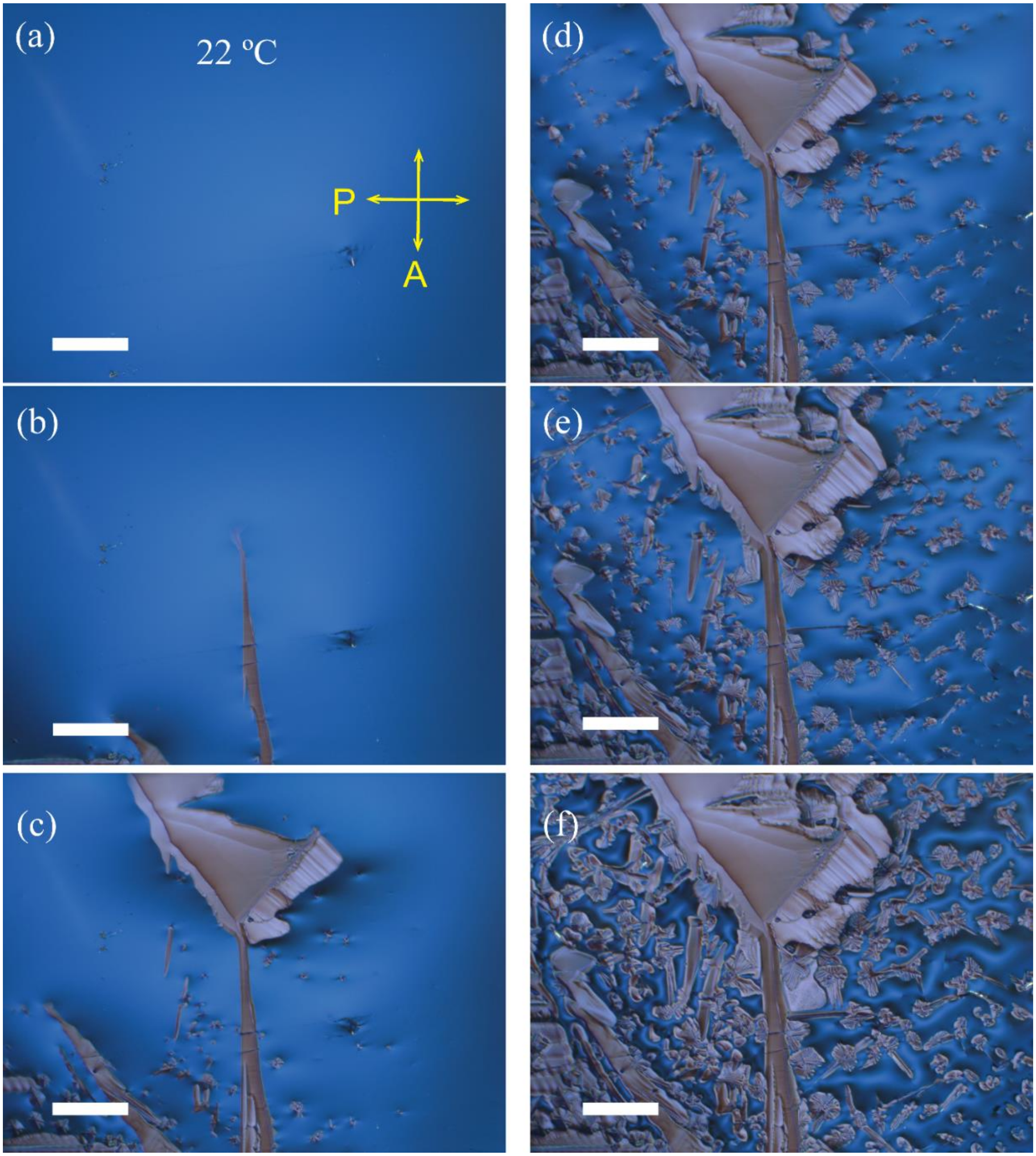

Figure S2. Open-air crystallization of bulk liquid Ga. Reflective polarized optical microscope (R-POM) image sequence during crystallization from the bulk liquid (a). Independent of the amorphous surface oxide, the crystallization of Ga proceed through the formation of crystalline Ga phase across the liquid surface (b-f). Scale bar: 400 µm.

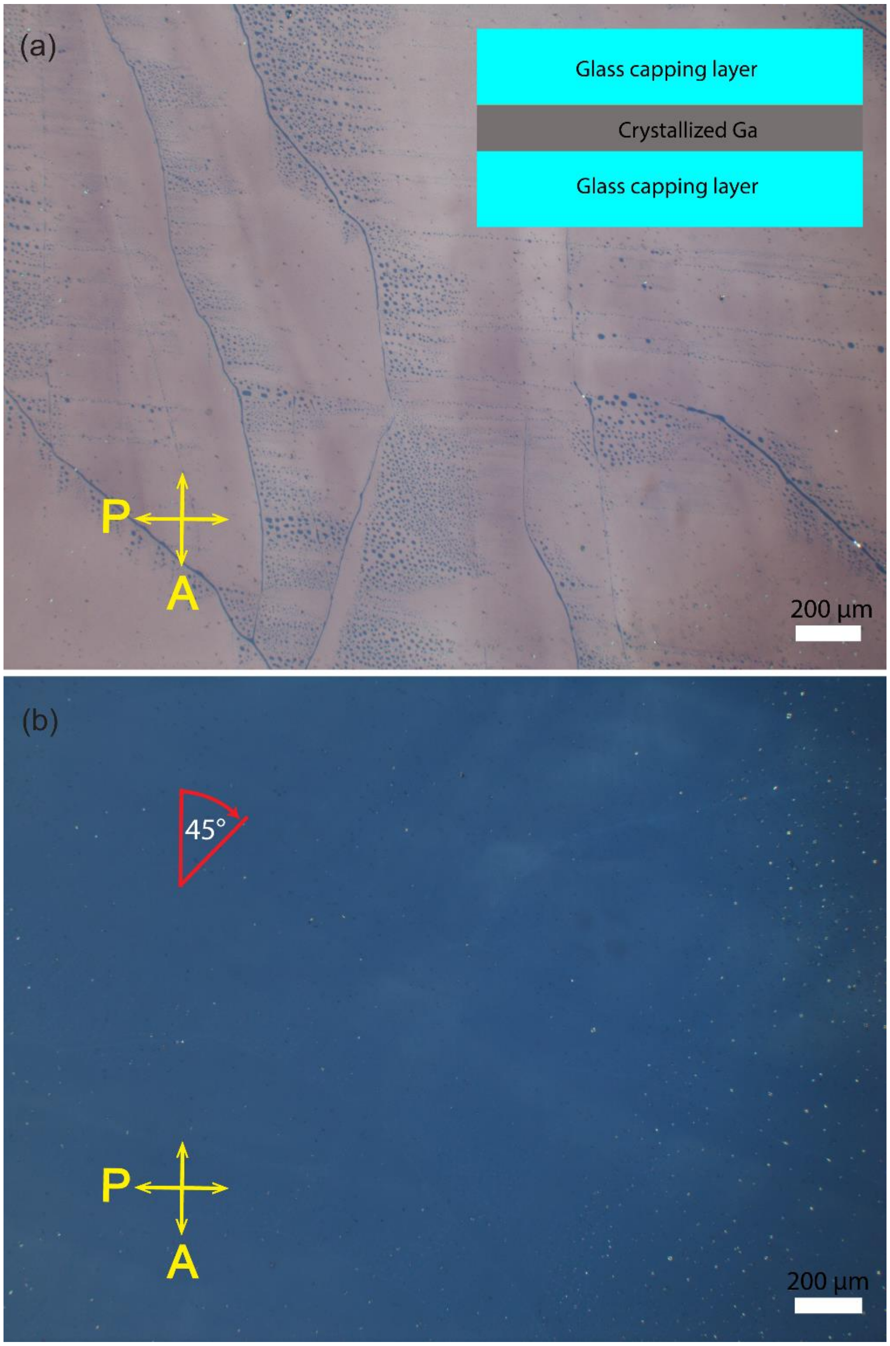

Figure S3. Large-area Ga crystal formation between glass slides in Movie S1. The crystallization is a first order transformation. (a) Crossed-polarized R-POM of the crystallized Ga with large are highly oriented domains. (b) Cross-polarized R-POM of the highly oriented crystallized Ga rotated 45º.

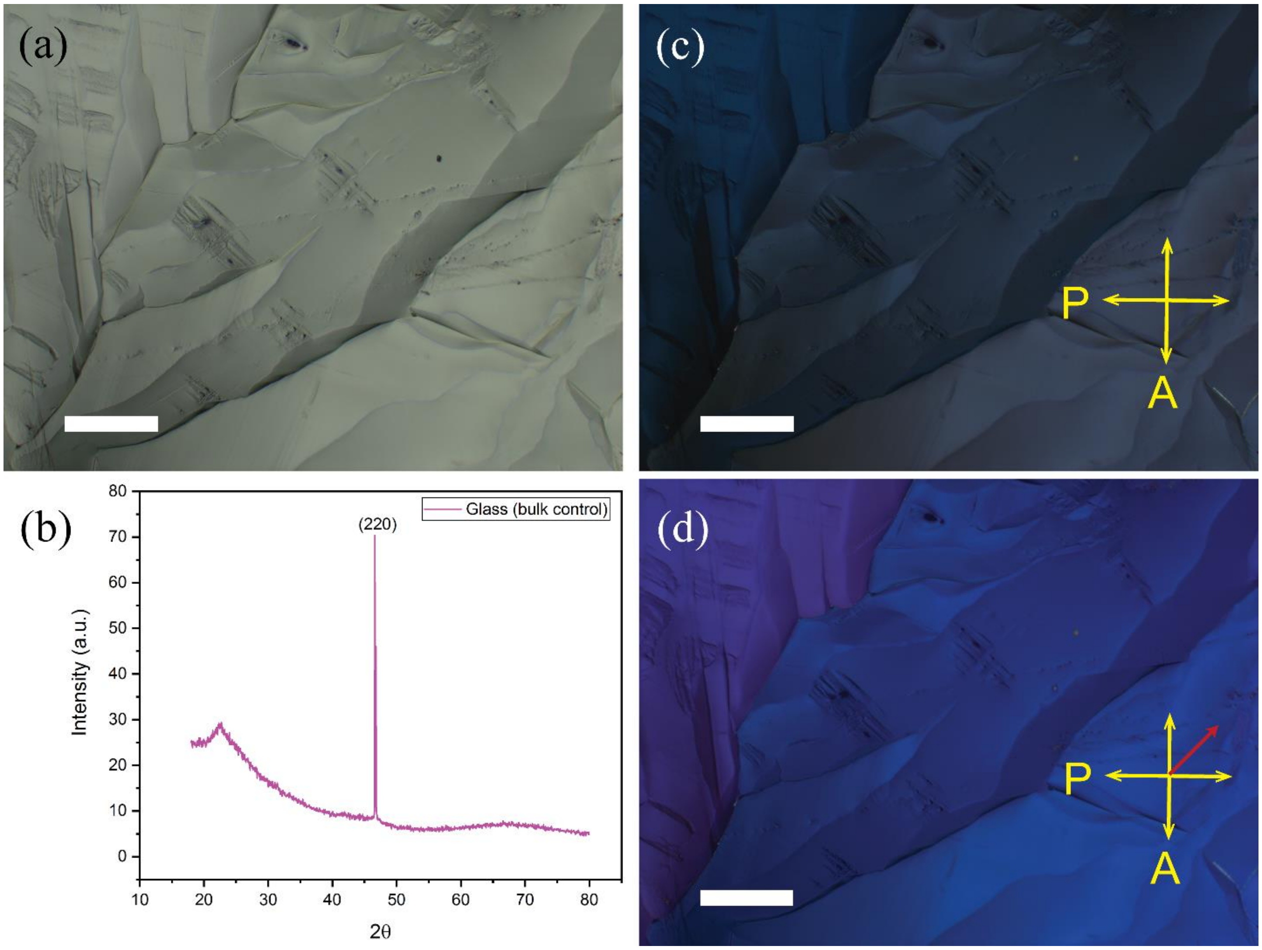

Figure S4. Bulk crystal of Ga obtained in ambient open-air conditions. (a) Bright field optical image of bulk Ga crystal with different domain orientation. (b) X-ray diffraction data of the bulk crystal where the peak at 46.6º corresponds to (220). (c) Crossed-polarized R-POM micrograph of bulk crystal in (a). Crossed-polarized R-POM micrograph of bulk crystal with a lambda plated inserted. Scale bars: 400 µm.

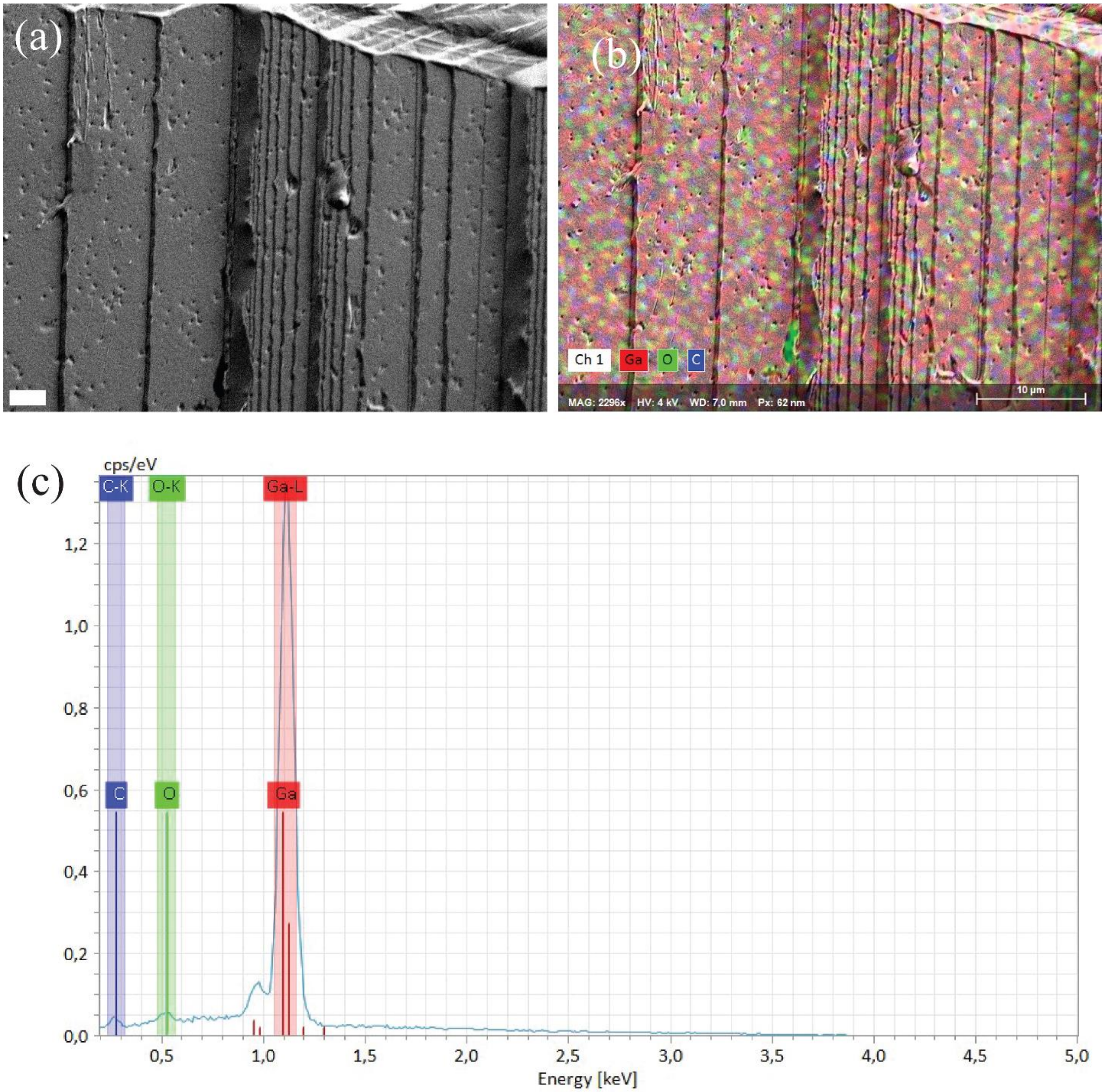

Figure S5. (a,b) Scanning electron microscopy (SEM) image and elemental analysis on the surface of the crystal Ga. (c) Energy-dispersive X-ray spectroscopy (EDS) analysis on bulk lamellar crystal of Ga.The energy spectra of the corresponding elemental composition on the Ga surface. Scale bar: 3 μm.

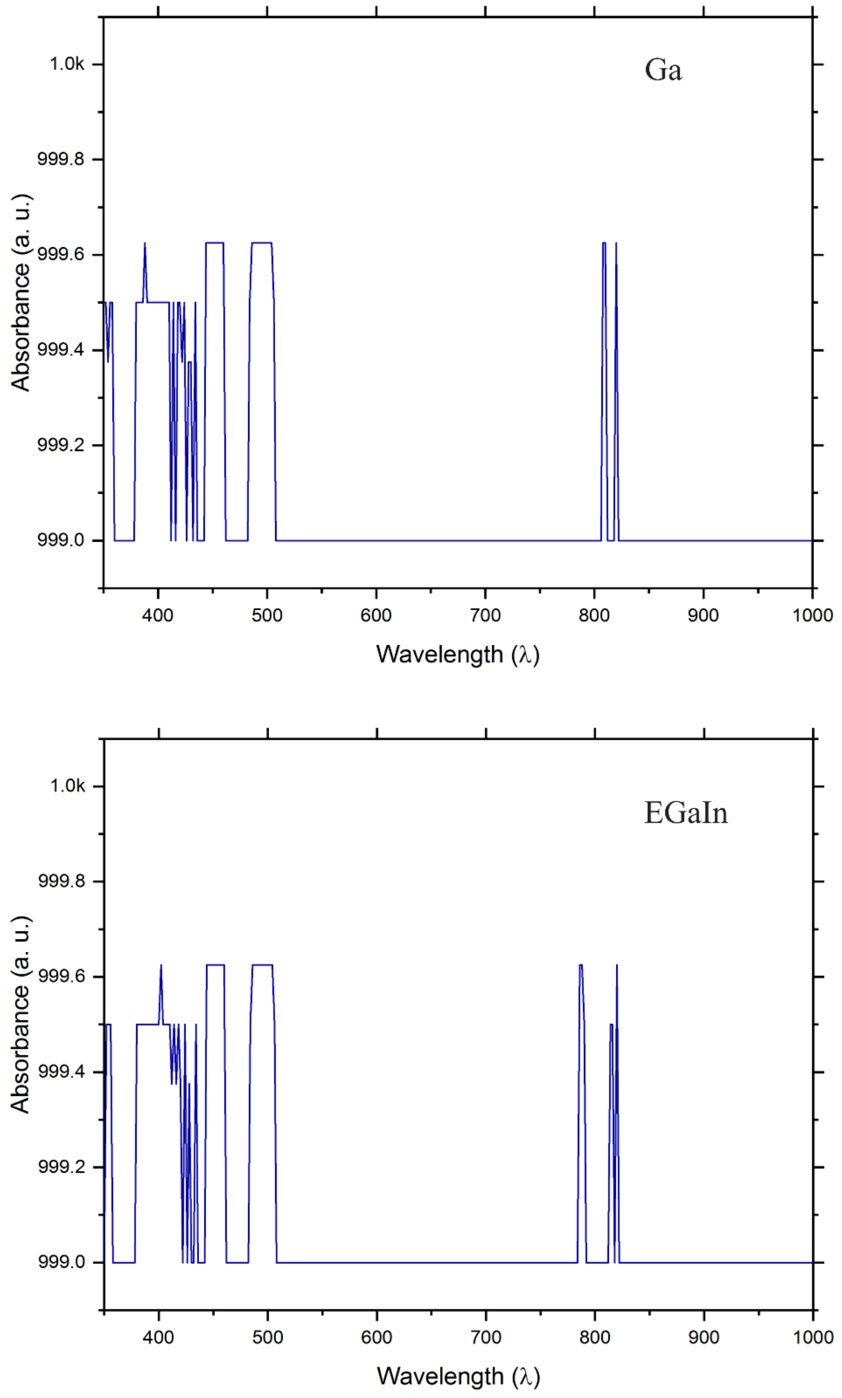

Figure S6. UV-Vis-NR absorption spectra of Ga and EGaIn with absorption at specific wavelengths.

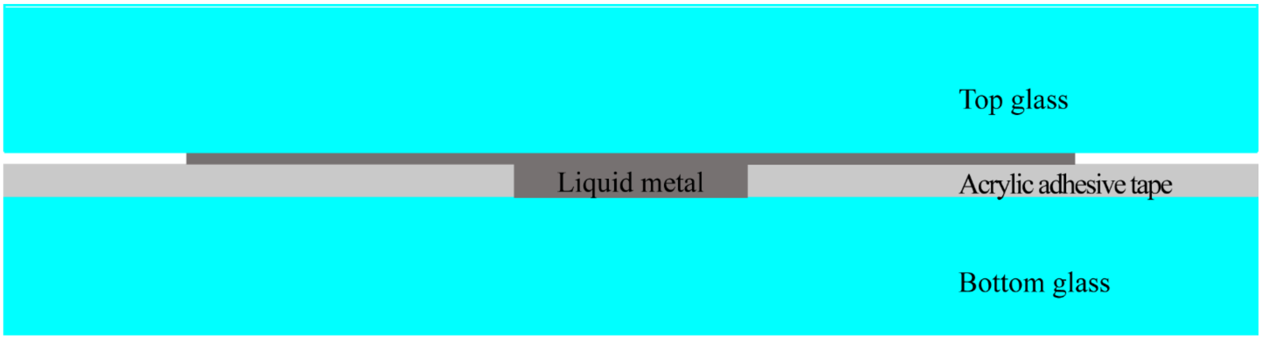

Figure S7. Schematic illustration of the flow-assisted dilation of the liquid Ga and EGaIn on an acrylic adhesive tape, where the liquid Ga is sheared between an untreated glass substrate and an acrylic coated glass substrate.

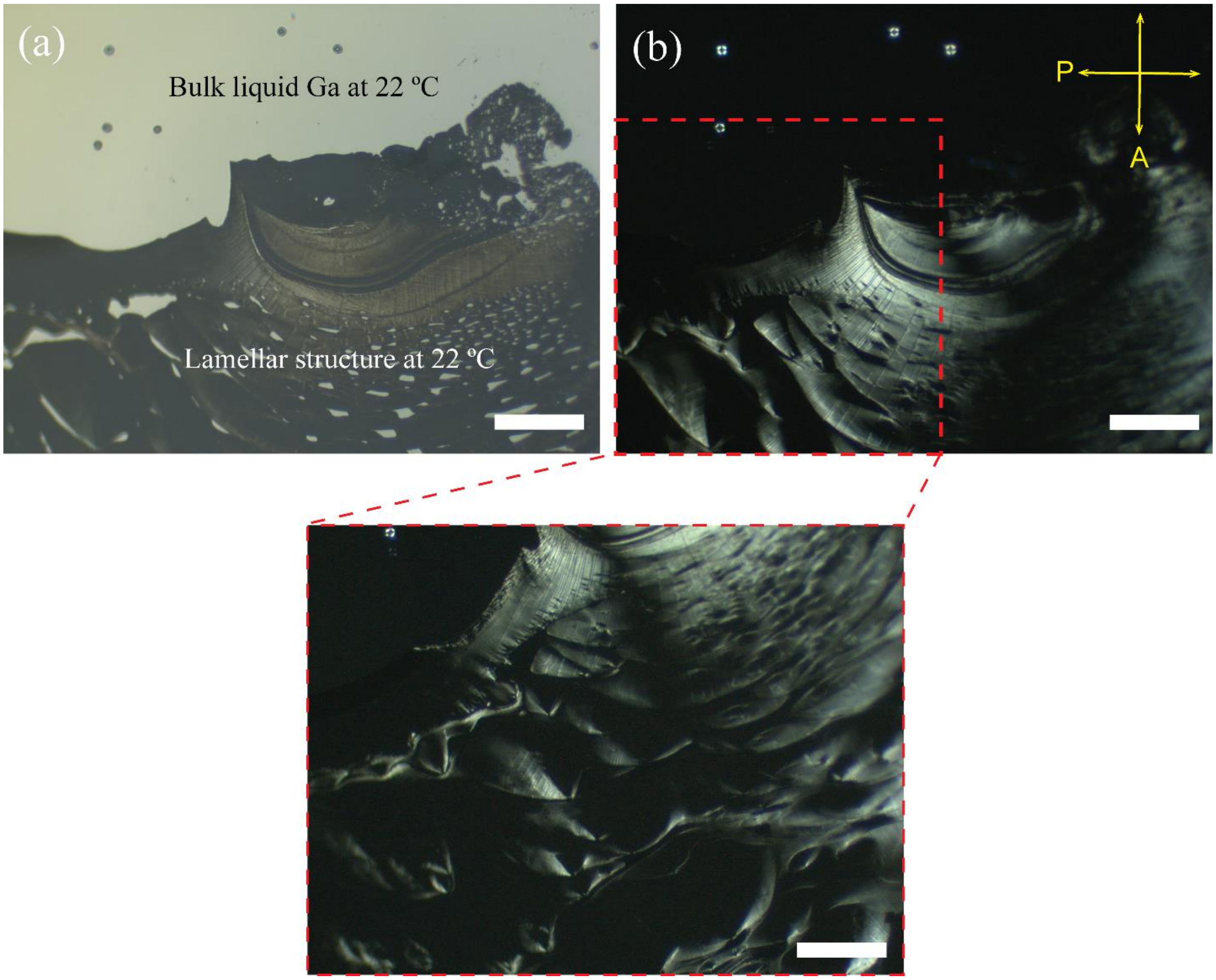

Figure S8. Lamellar orientation and parabolic focal conics (PFC) type domains. (a) Optical image of the bulk liquid and dilated lamellar structure. (b) Crossed-polarized R-POM image of the bulk liquid and dilated lamellar structure. Scale bar: 100 µm.

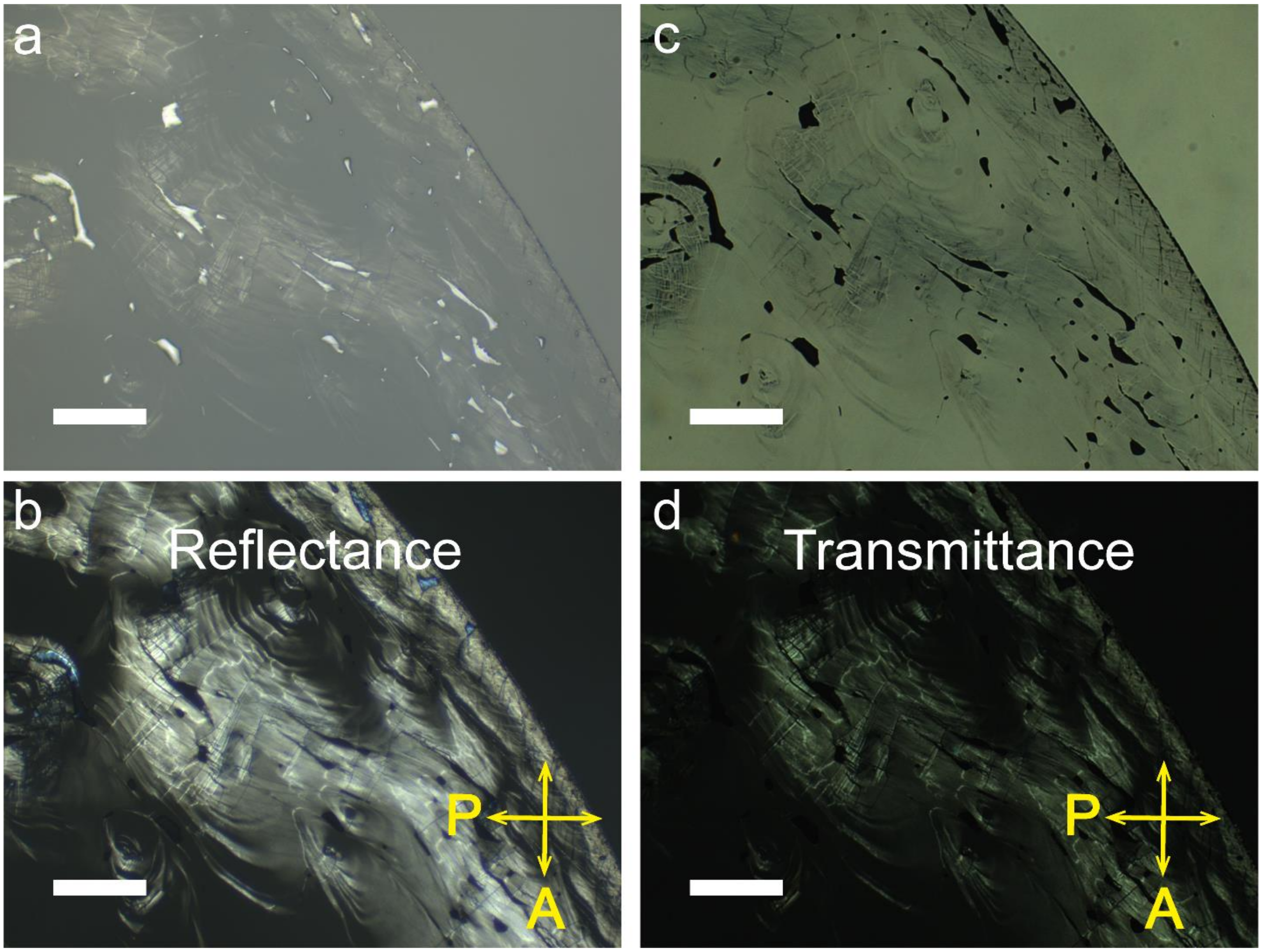

Figure S9. Additional POM texture of the liquid Ga sheared between an untreated glass surface and an acrylic coated glass surface depicting the horizontal domain texture. The same sample shown in brigh field without polarizers (a) and (c), crossed-polarized R-POM image (b), and transmission polarized optical microscopy (T-POM) image (d). Scale bars: 100 µm.

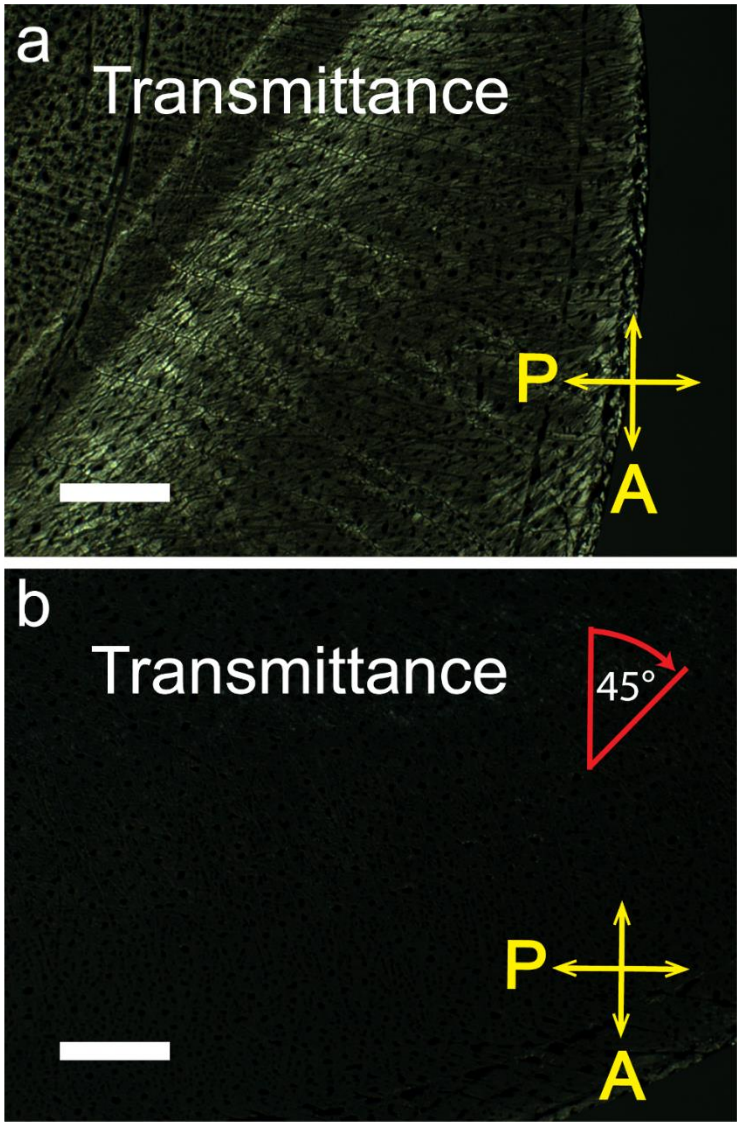

Figure S10. Additional T-POM texture image of the liquid Ga film sheared between an untreated glass surface and an acrylic coated glass surface with an unknown thickness. Crossed-polarized T-POM image (a) and crossed-polarized T-POM image with the sample rotated 45º with respect to the polarizers (b). Scale bar: 100 µm.

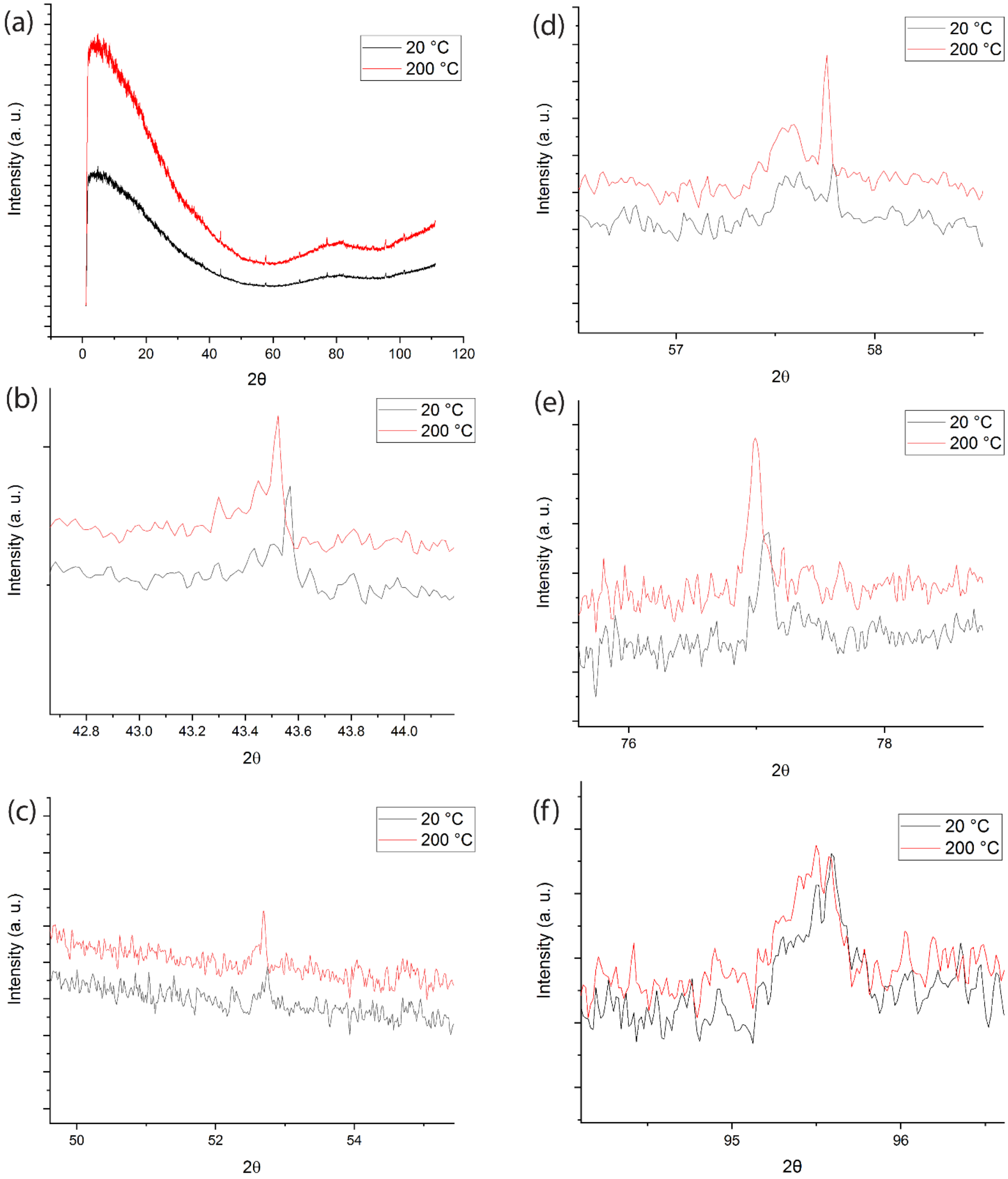

Figure S11. X-ray diffraction (XRD) patterns of the liquid Ga taken at 20 °C and 200 °C. (a) The diffraction of liquid Ga capillary shows sharp reflection and hump in the background. The sharp reflection comes from nanoclusters suspended in the supercooled liquid background. Upon heating, the XRD patterns shifted. (b), (c), (d), (e), and (f) are the enlarged peaks from (a) at 2θ values of 43.5º, 52.7º, 57.8º, 77º, and 95.5º. The corresponding *d* spacing are 2.08, 1.74, 1.59, 1.24, and 1.04 Å at 200 °C. All other peaks below 40º are missing.

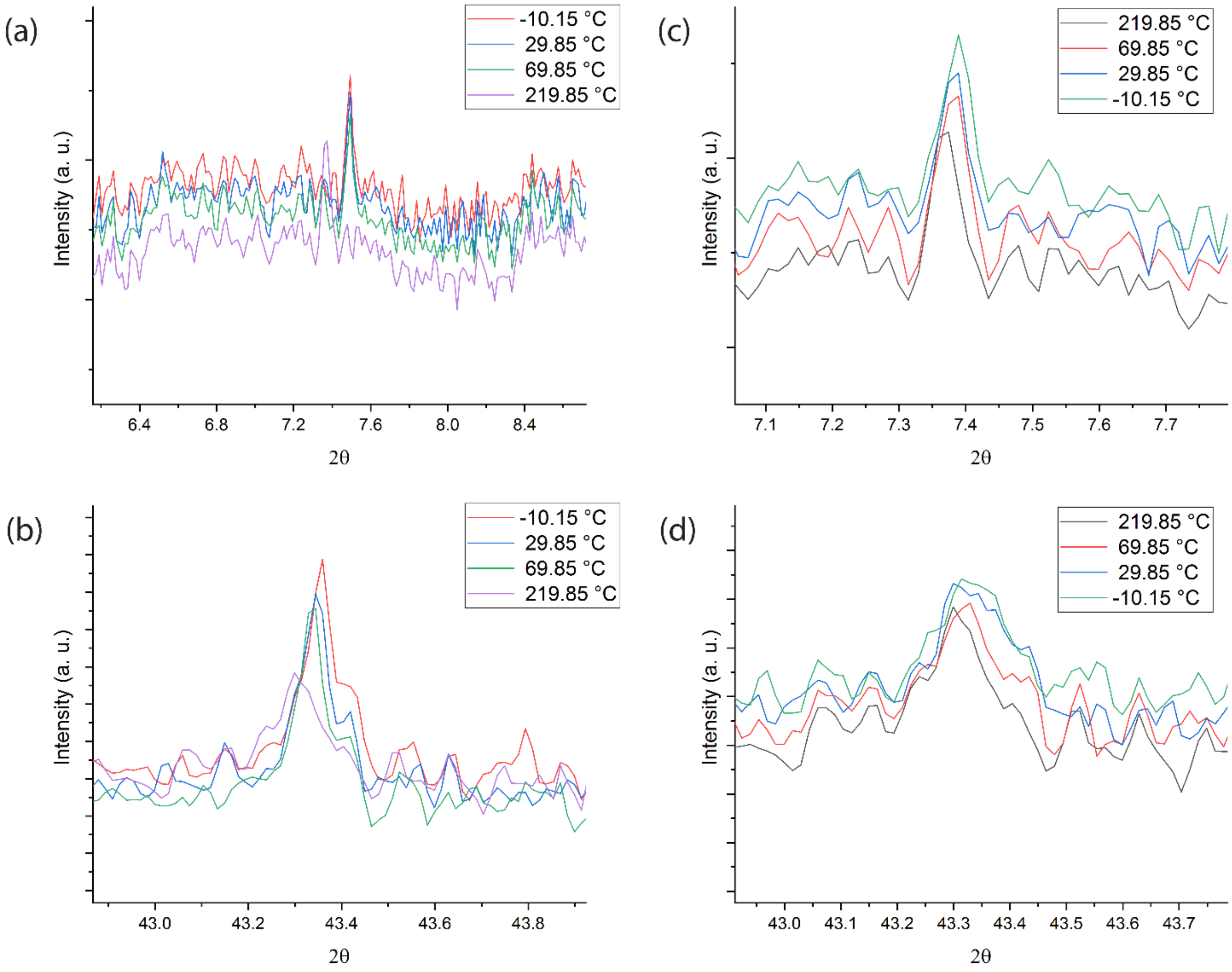

Figure S12. XRD patterns of liquid Ga taken at subsequent temperatures during heating and cooling. (a,b) heating from -10.15 °C (263 K) to 219. 85 °C (493 K) and (c,d) cooling from 219.85 °C to -10.15 °C.

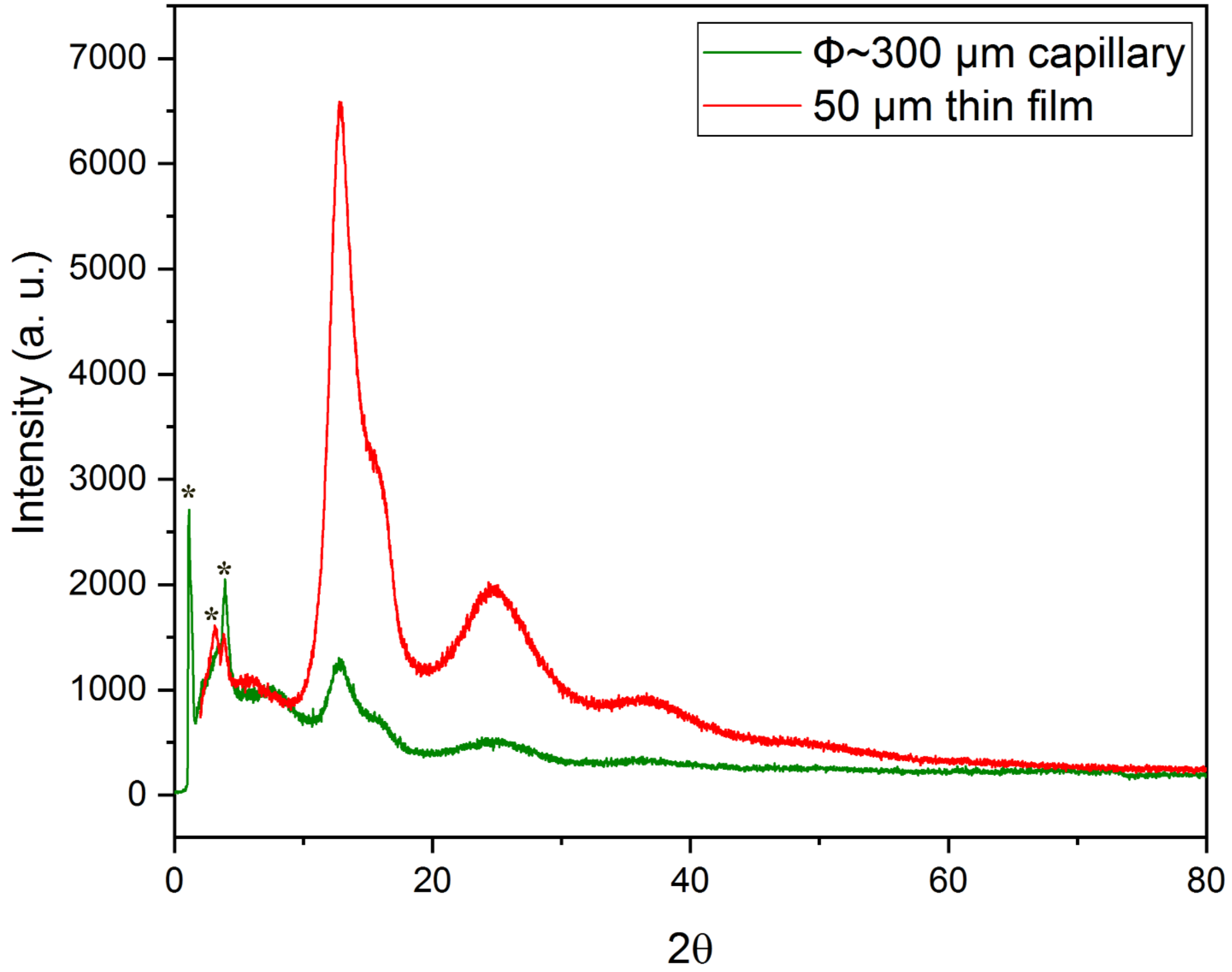

Figure S13. XRD patterns of the Ga capillary and glassy film. A supercooled liquid gallium in capillary with 300-µm diameter (green) and slowly cooled glassy 50-µm Ga film (red) show a typical amorphous supercooled structure of supercooled liquid Ga. The X-ray scattering intensity was obtained in transmission using Ag-Kα1 radiation (0.56 Å). Unlike in the Cu- Kα1 diffraction, there are no wide-angle sharp peaks detectable when Ag radiation is used. The peak (*) is due to device artifact.

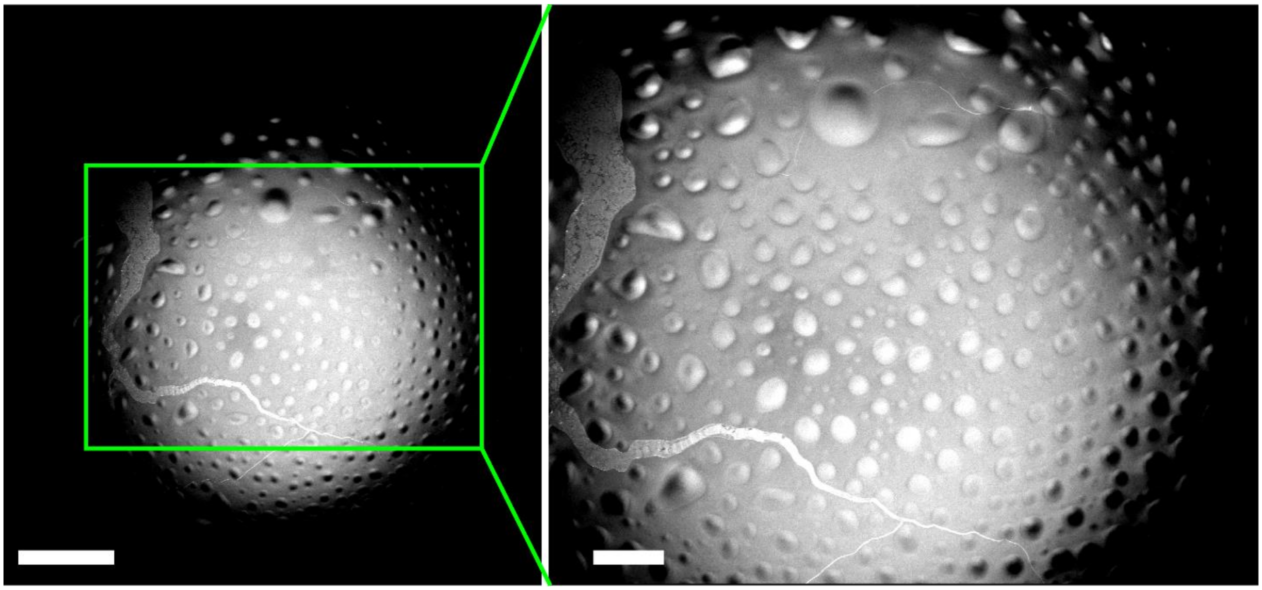

Figure S14. SEM micrographs of the liquid Ga droplet. The surface of the droplet was cleaned in 0.5 M HCl solution to remove any oxide before SEM imaging. The SEM of the Ga droplet shows structures similar to focal conic domains of smectic droplets. Scale bars: (a) 100 µm and (b) 40 µm. Droplet diameter is around 600 µm.

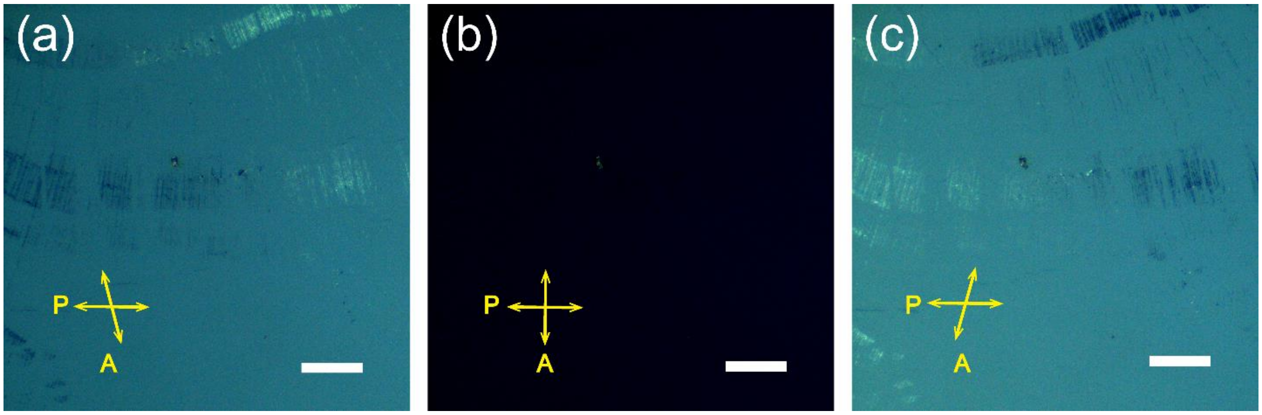

Figure S15. R-POM images of the homeotropic texture of the slowly cooled (2 °C/min) liquid Ga film between polyimide-treated glass plates with a cell gap of 17 µm. (a,c) Uncrossing one of the polarizers by ±6°. (b) Cross-polarized R-POM image. Scale bars: 200 µm.

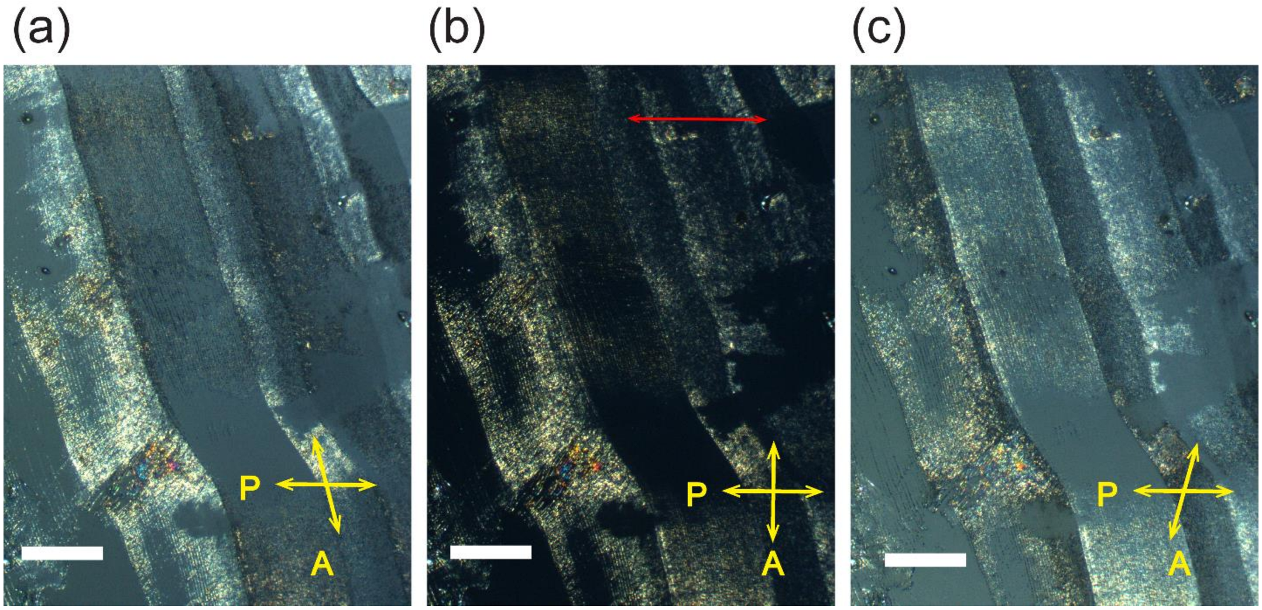

Figure S16. R-POM images of the texture of the liquid Ga slightly sheared between clean glass plates with a cell gap of 10 µm. When sheared, large domains splitting into brighter and dark bands following the uncrossing of the polarizers. (a,c) Uncrossing one of the polarizers by ±4º. (b) Cross-polarized R-POM. Red arrow represent the shear direction. Scale bars: 100 µm.

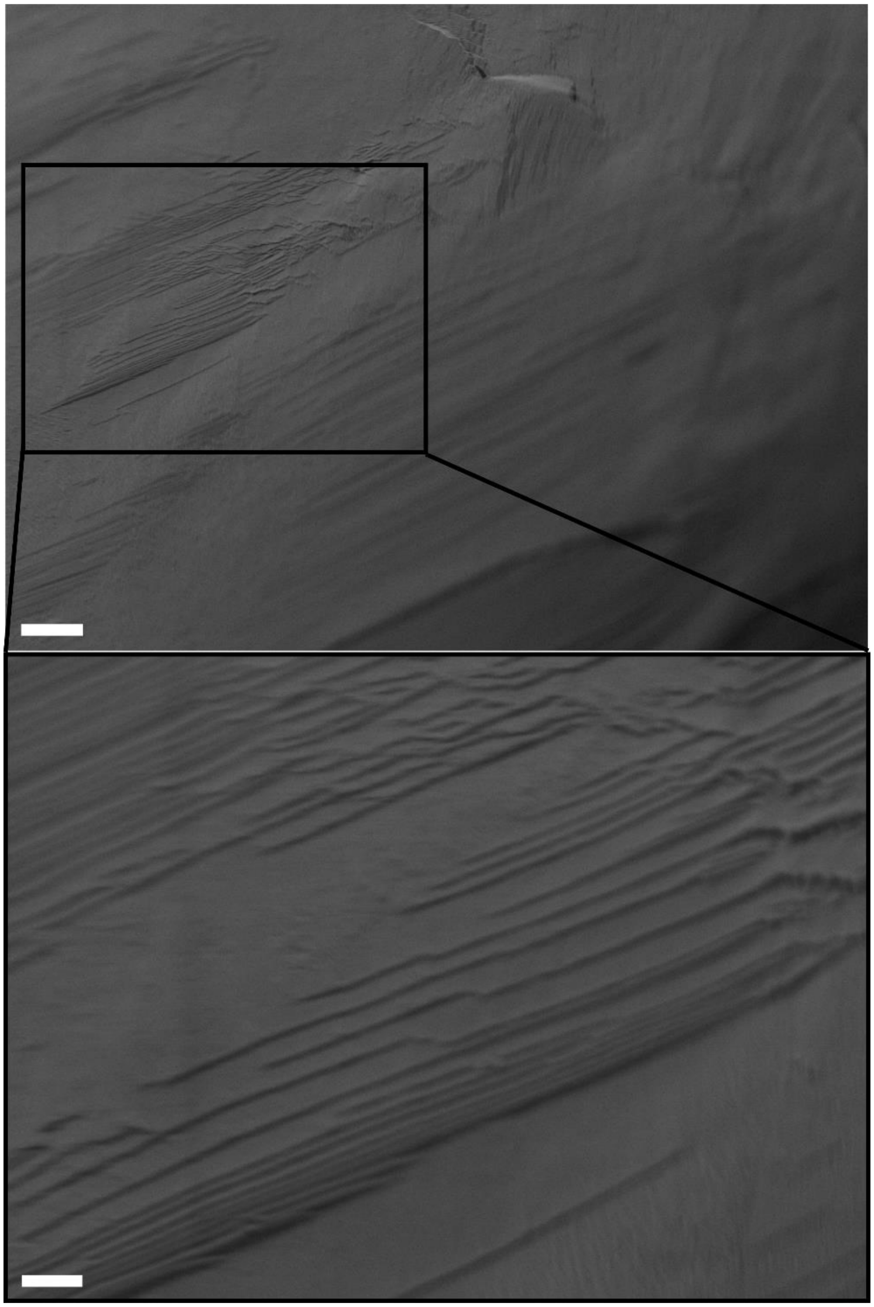

Figure S17. Additional SEM micrographs of the lamellar steps in bulk film of the solid Ga. Scale bars: 2 µm and 400 nm in the rectangular inset.

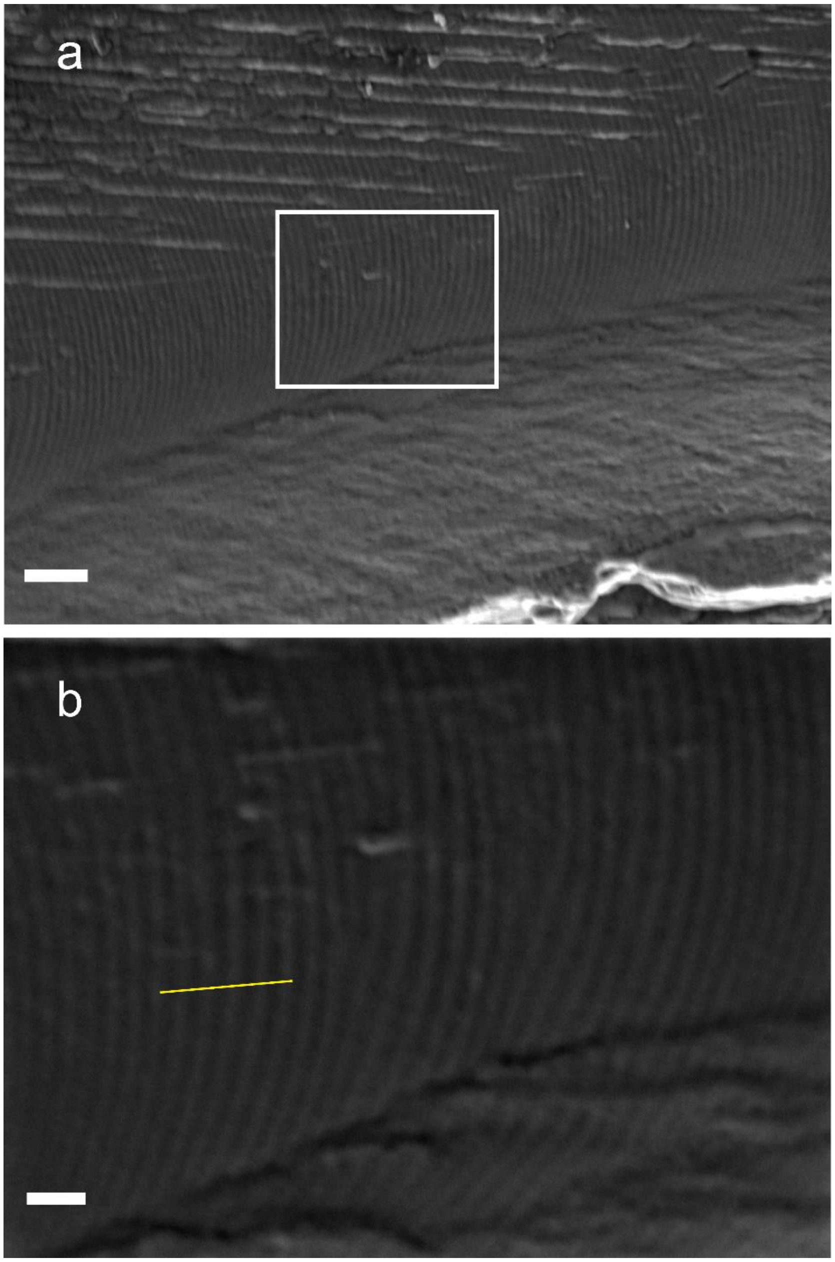

Figure S18. SEM micrographs of the slowly cooled Ga in a capillary tube (~1 mm diameter) reveals stripe structures with thickness of 40 nm. Images were taken from the bulk region after fractured in liquid nitrogen. Scale bars: (a) 300 nm and (b) 100 nm rectangular region in (a).

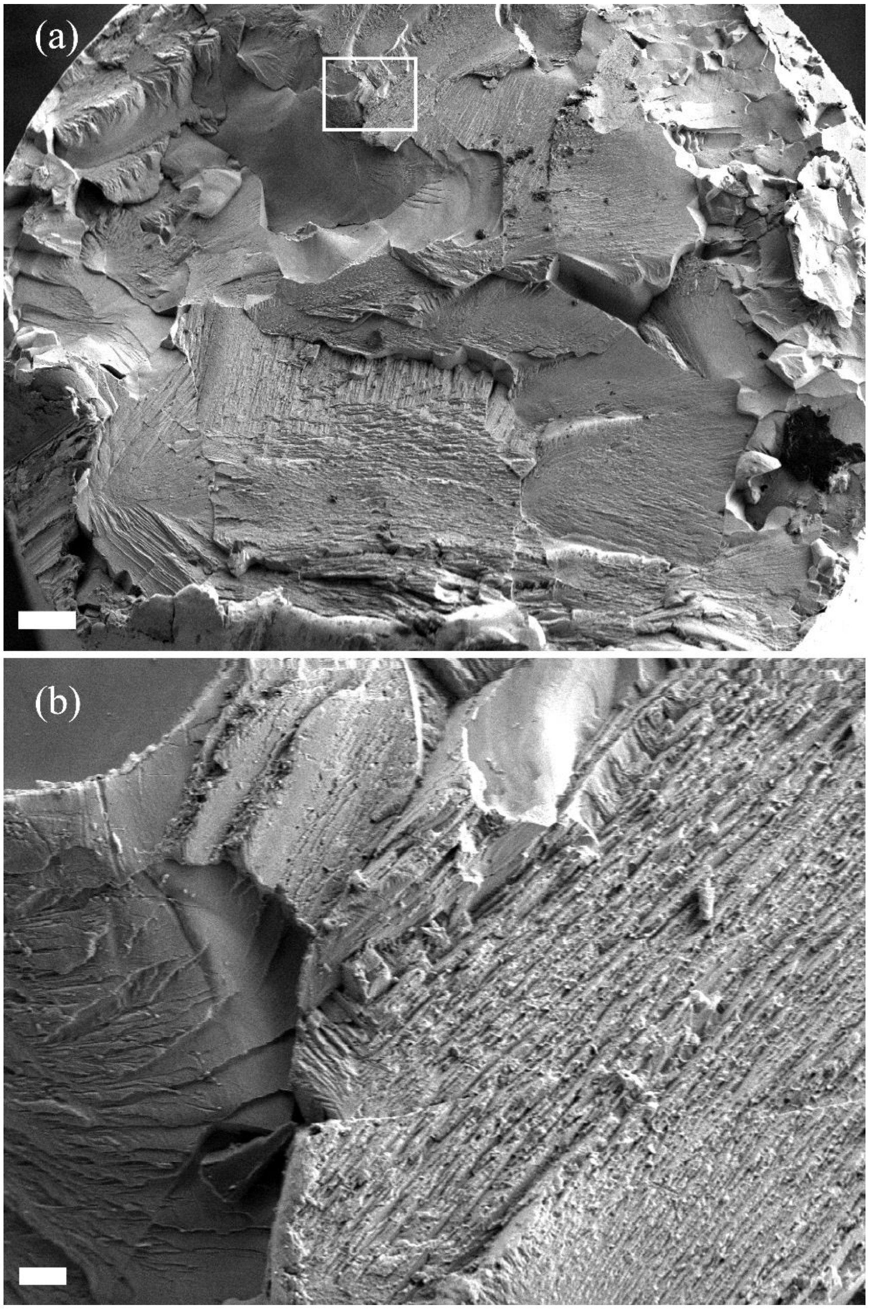

Figure S19. SEM micrographs of the quenched Ga in a capillary tube (0.5 mm diameter). Scale bars: (a) 30 µm and (b) 2 µm.

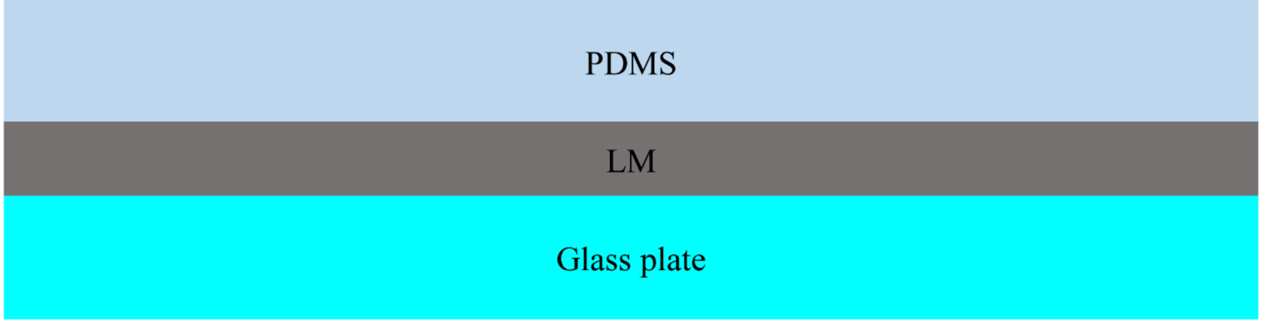

Figure S20. Schematics of the exfoliation method. In a sandwich configuration with different anchoring condition, liquid metal (LM) assumed planar alignment near the PDMS surface. Upon the separation of the upper PDMS layer, the lamellar structure becomes apparent in the T-POM images.

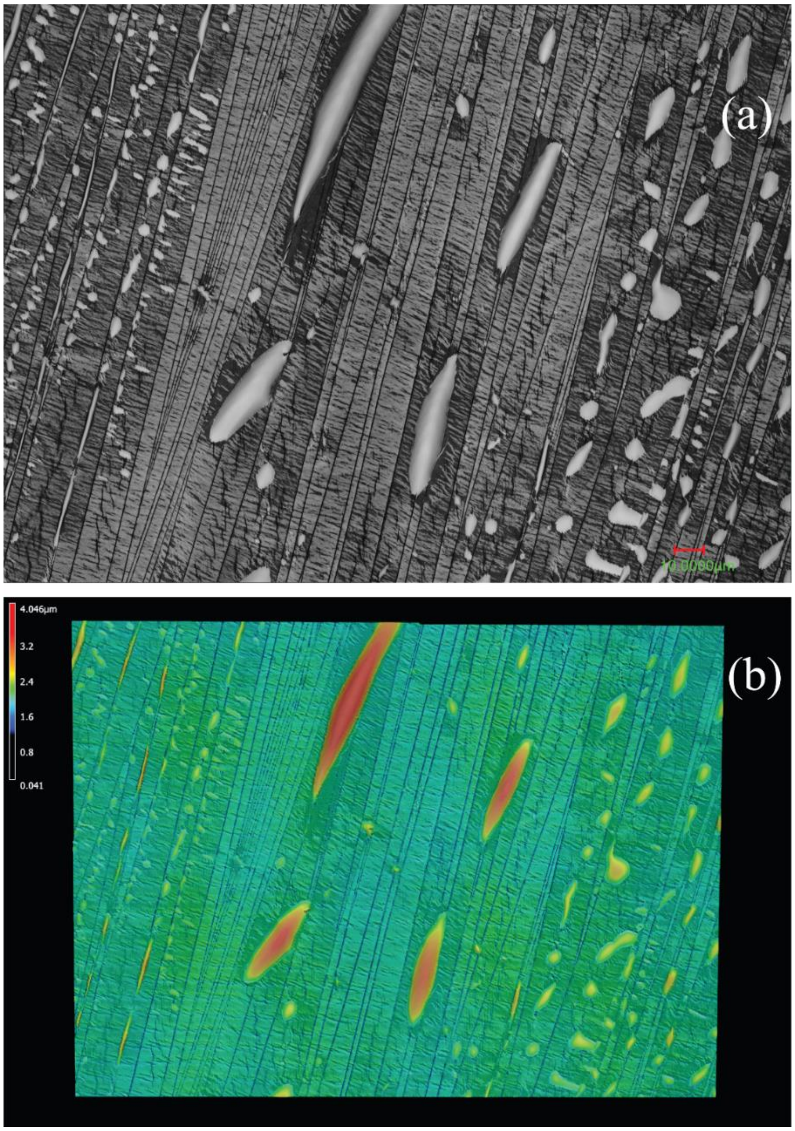

Figure S21. Confocal 3D laser microscope investigation of the exfoliated lamellar structure on PDMS surface. (a) Optical image and (b) height representation of surface topography. Liquid metal residue are shown as the highest feature on the surface as seen in (b).

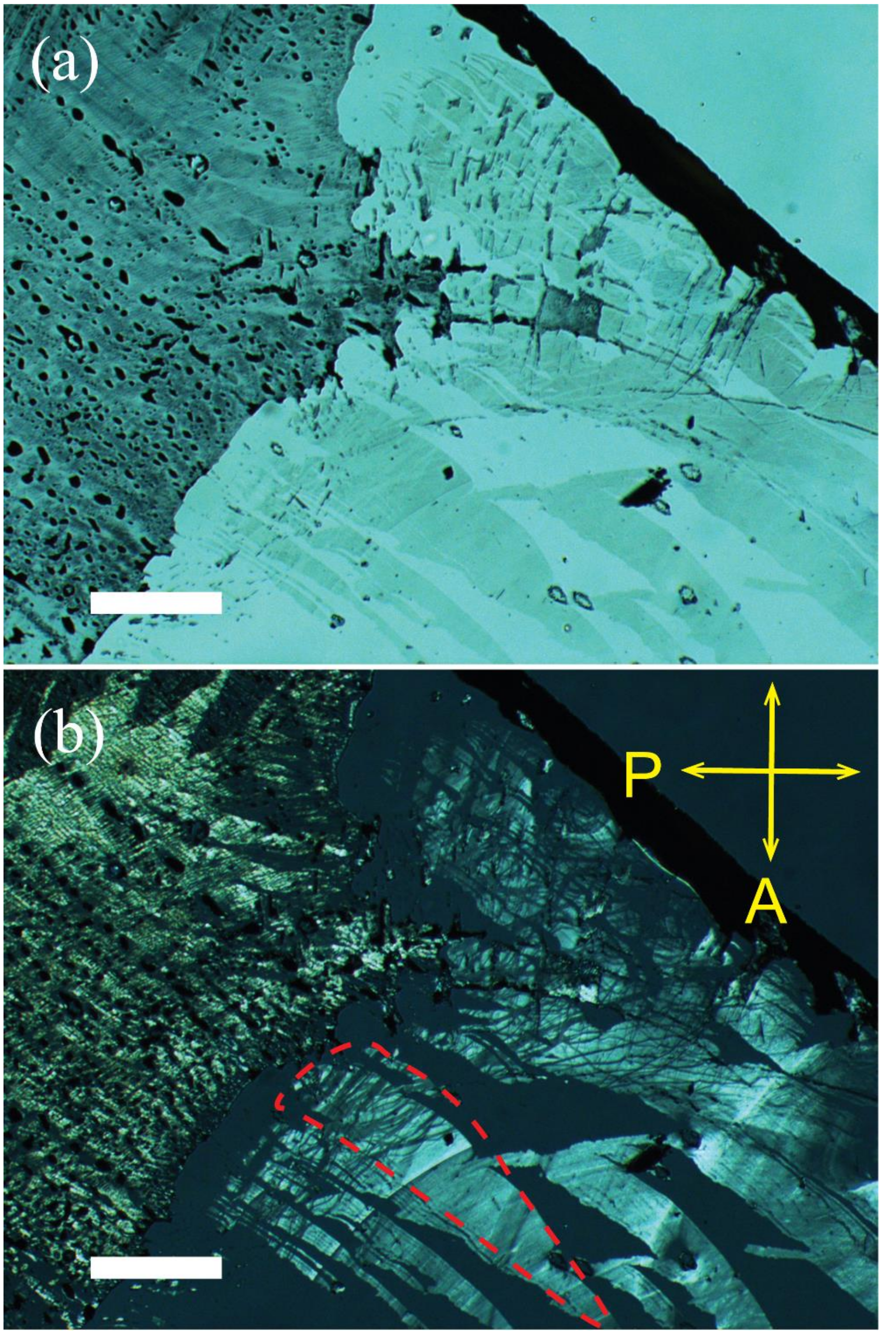

Figure S22. Additional T-POM images of the exfoliated lamellar structure. (a) Bright field and (b) crossed-polarized T-POM images. The red area shows a large lamellar area. Scale bars: 100 µm.

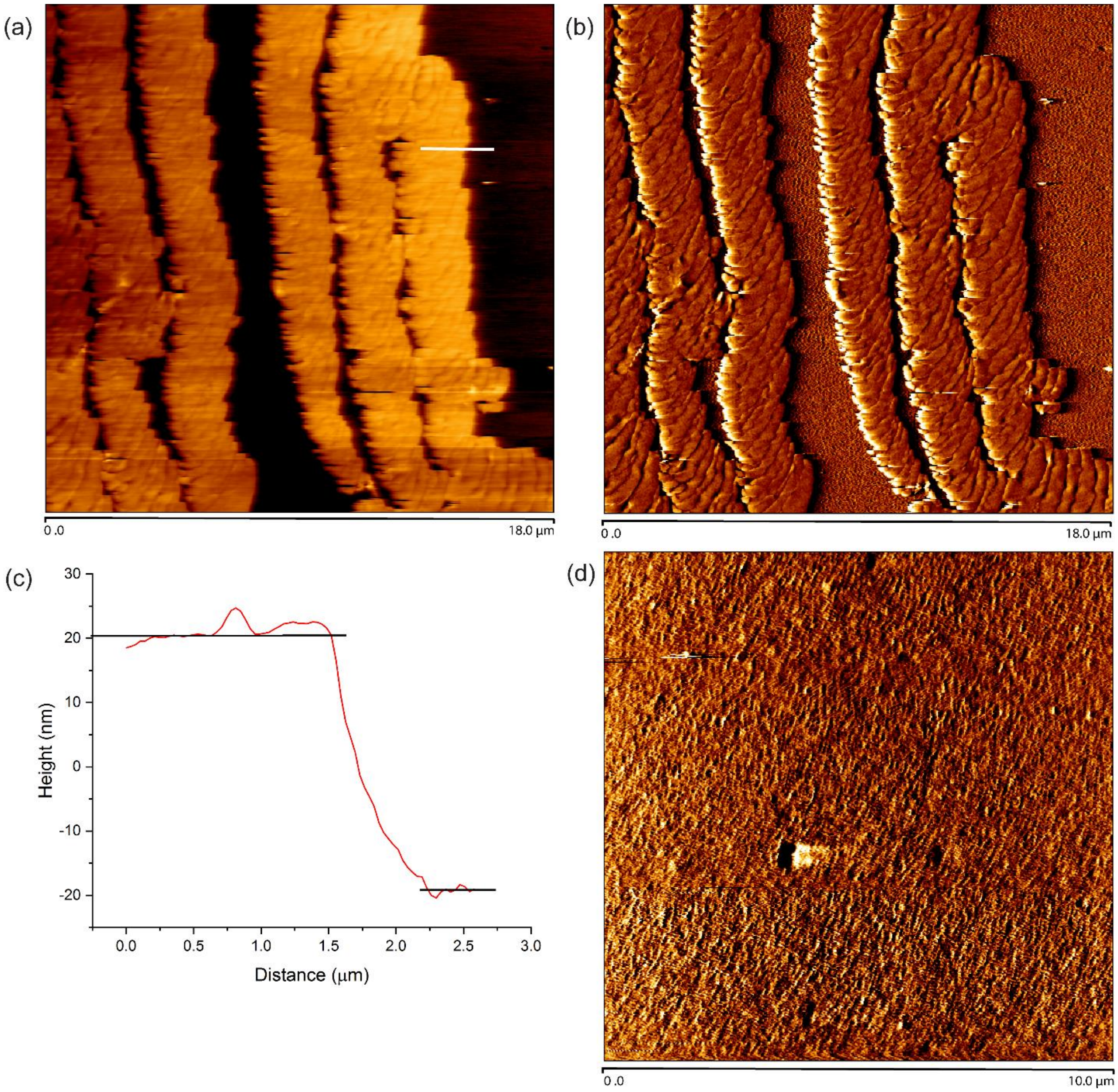

Figure S23. Additional atomic force microscopy (AFM) topology of the exfoliated lamellar structure surface. (a) Height image and (b) vertical deflection of a single layer structure. (c) Corresponding height measurement of line analysis in (a). (d) Reference PDMS substrate AFM topography.

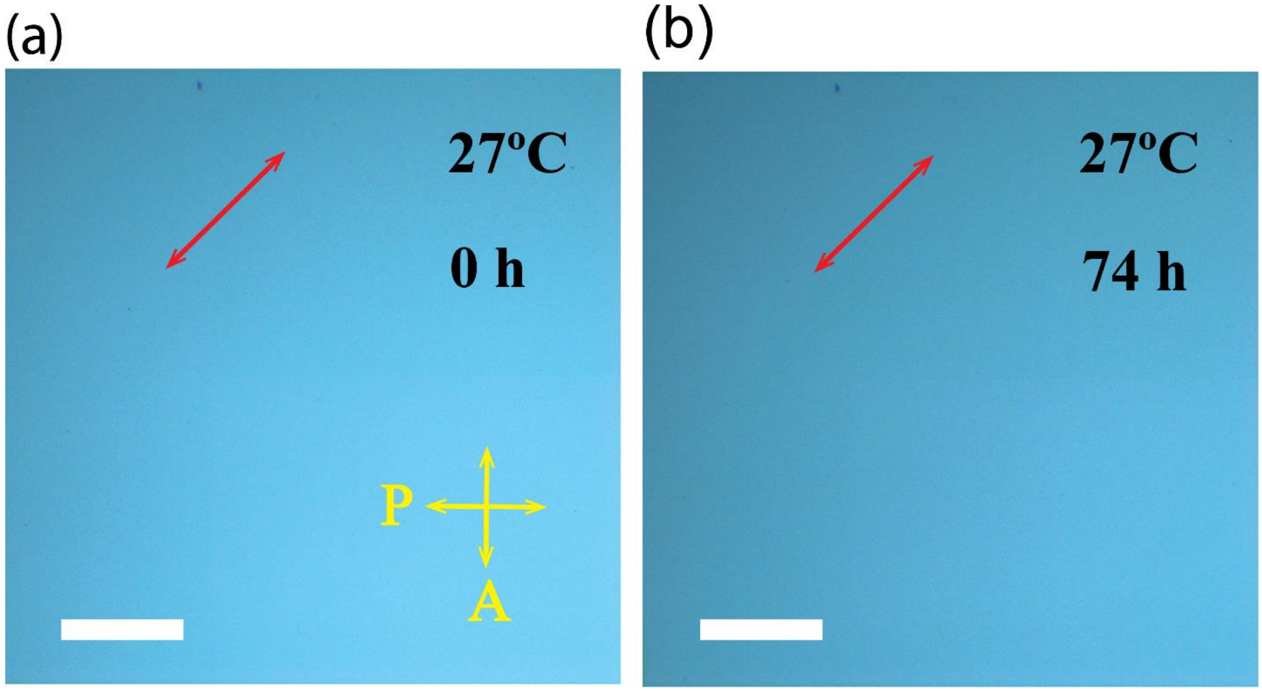

Figure S24. Stability experiment of the supercooled liquid Ga film in 10-µm thick planar cell confinement. R-POM images at 27 ℃ over 0 h (a) and 74 h (b). Scale bars: 100 µm.

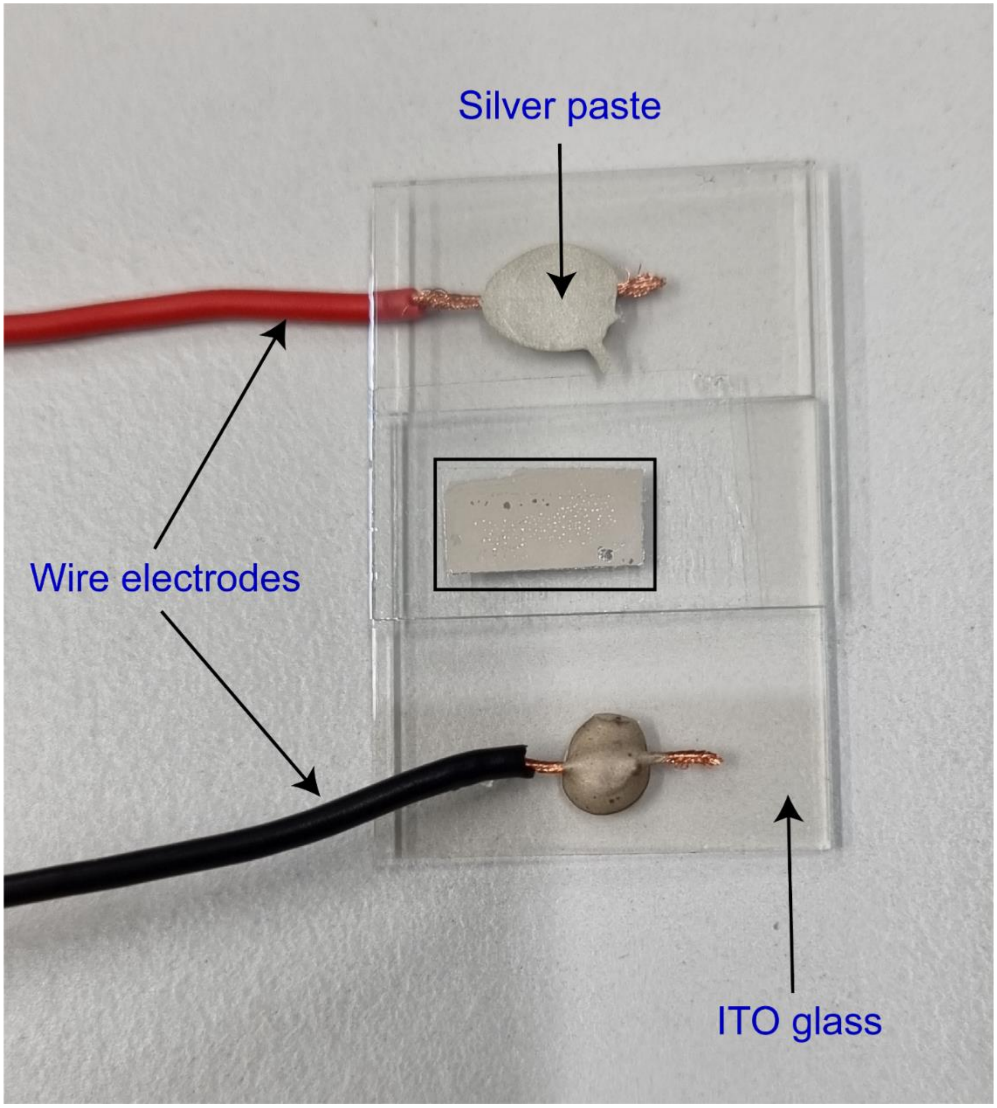

Figure S25. Photo of the electro-optic device assembly. The black area in the image shows the silvery liquid metal sample sandwiched between two ITO glasses.

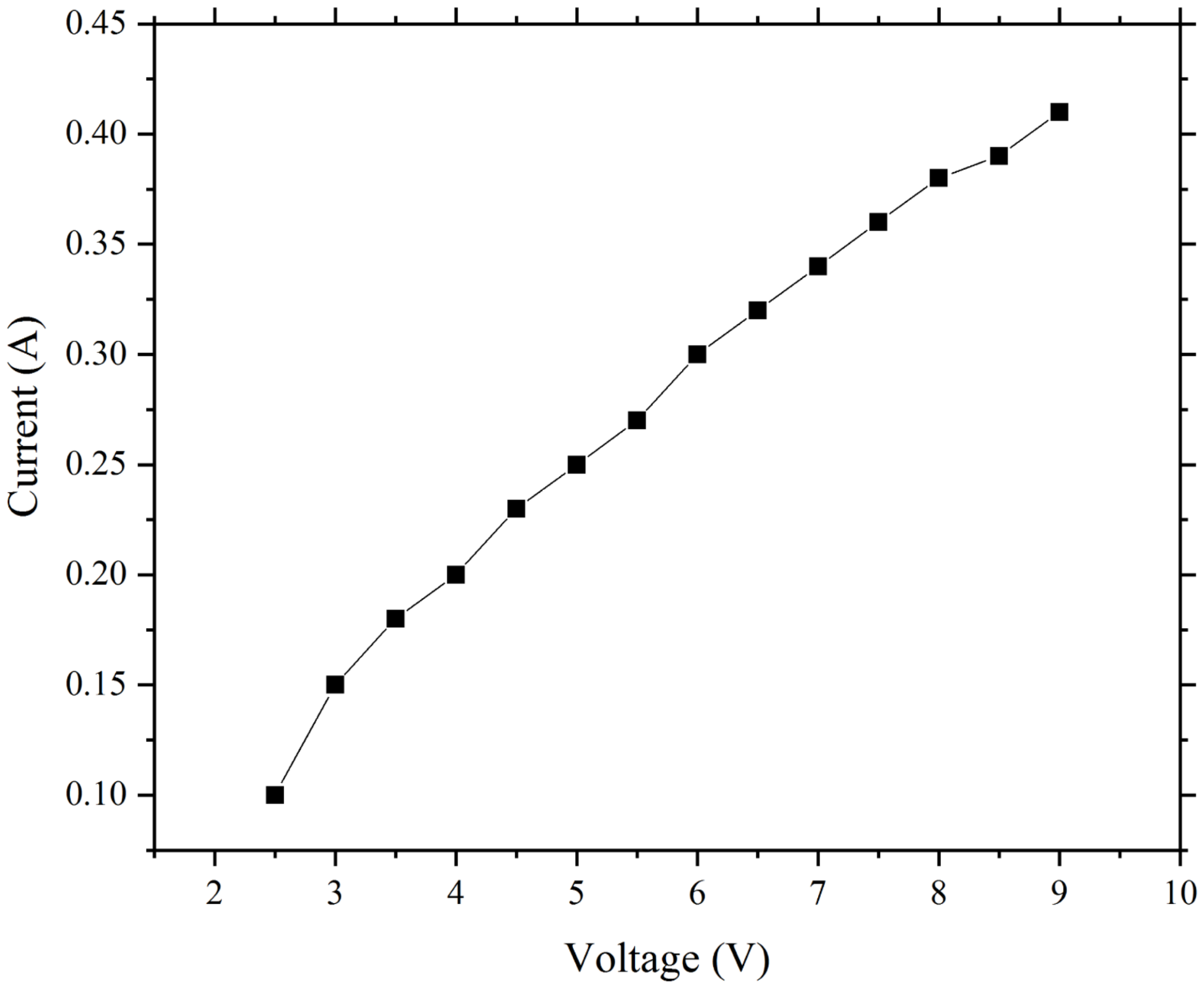

Figure S26. Current-voltage response across the Ga film confined in a LC cell.

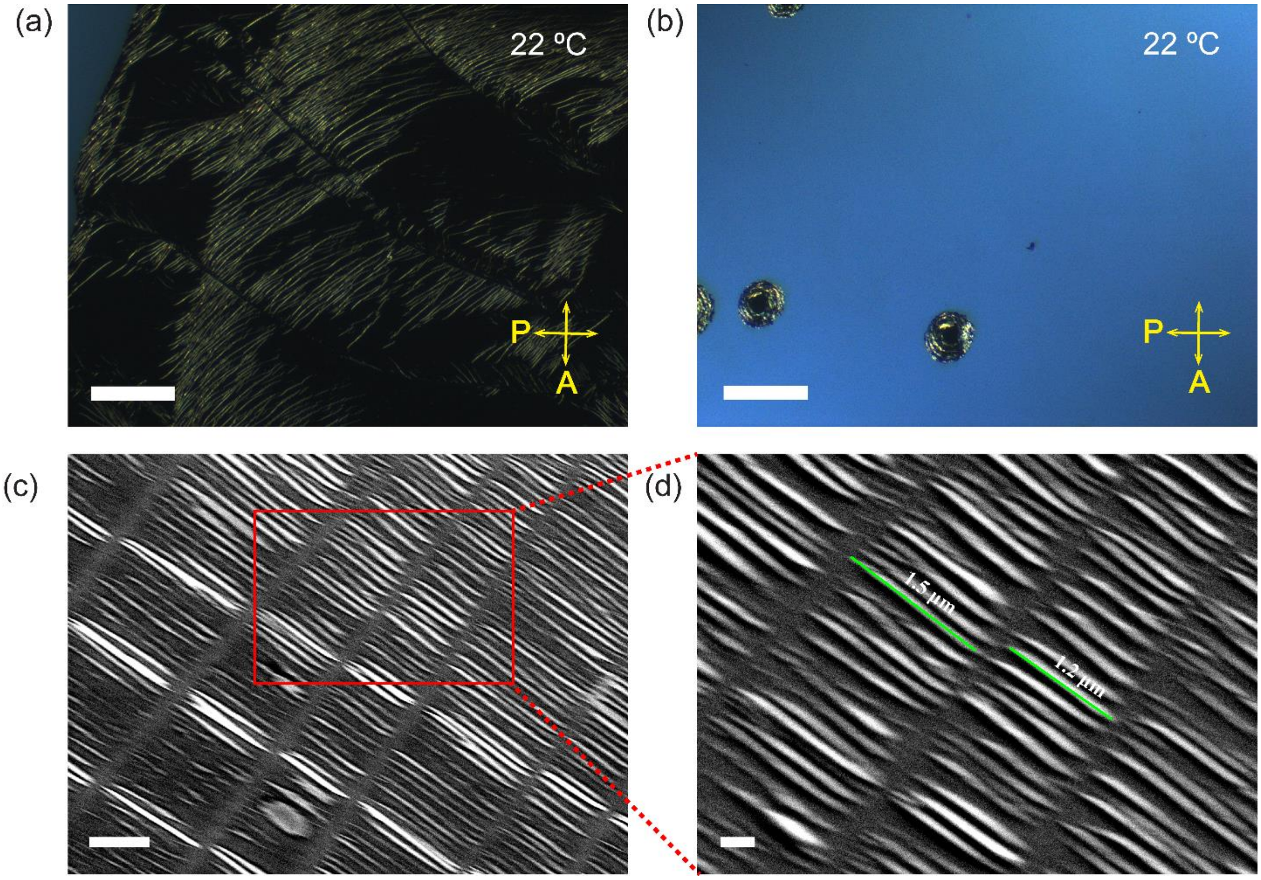

Figure S27. Stripe texture formation at temperatures above 120 °C. R-POM images of stripes in (a) Ga and (b) EGaIn at 22 °C. The cells were cooled to 22 °C from 145°C. SEM image of the submicron-needles with periodicity of about 100 nm in EGaIn (c) and enlarged rectangular area texture (d). The texture was stabilized at room temperature prior to SEM imaging. Scale bars: (a) and (b) 100 µm, (c) 1 µm, and (d) 300 nm (inset).

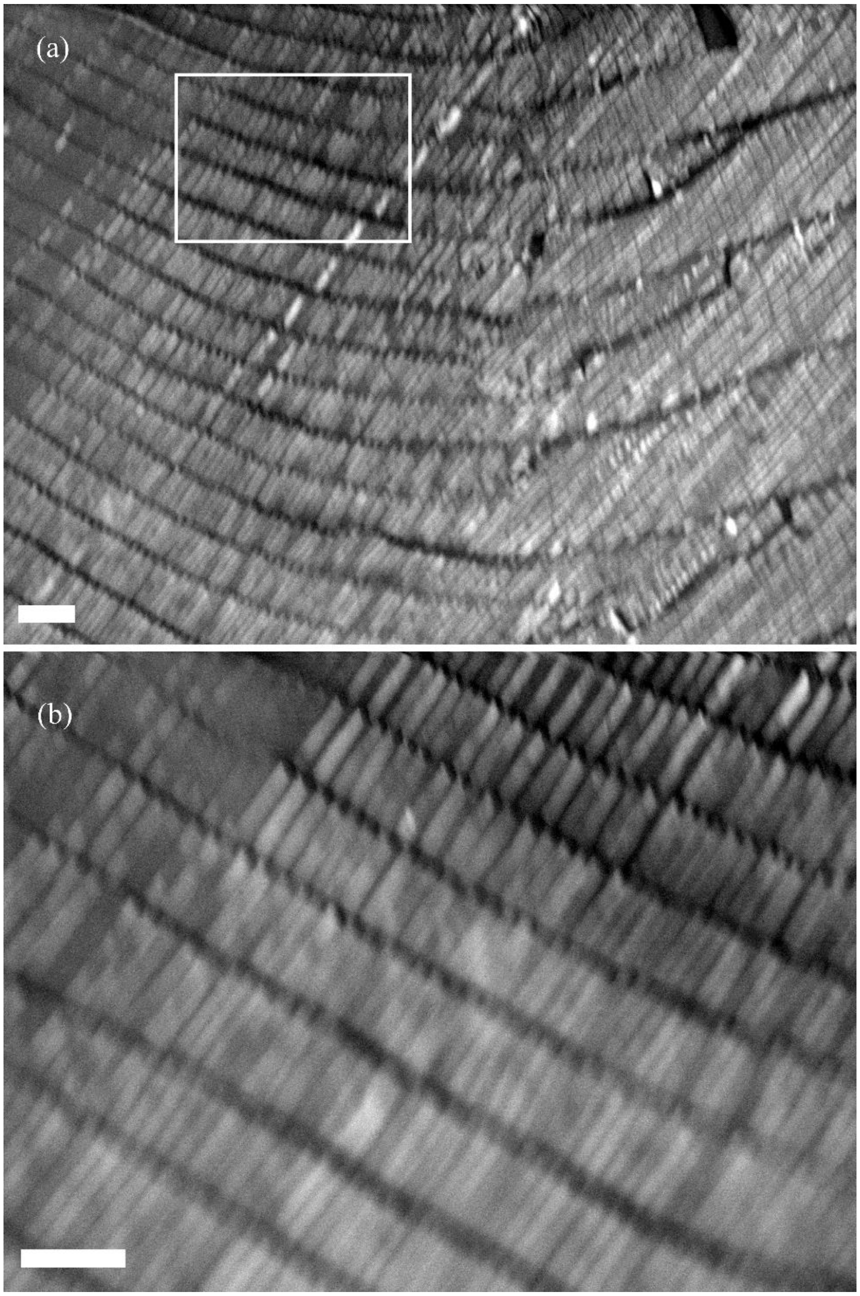

Figure S28. SEM images showing spatial arrangement of lamellar or needle-like structures near birefringent bubble in homeotropic cell. Scale bars: 1 μm.

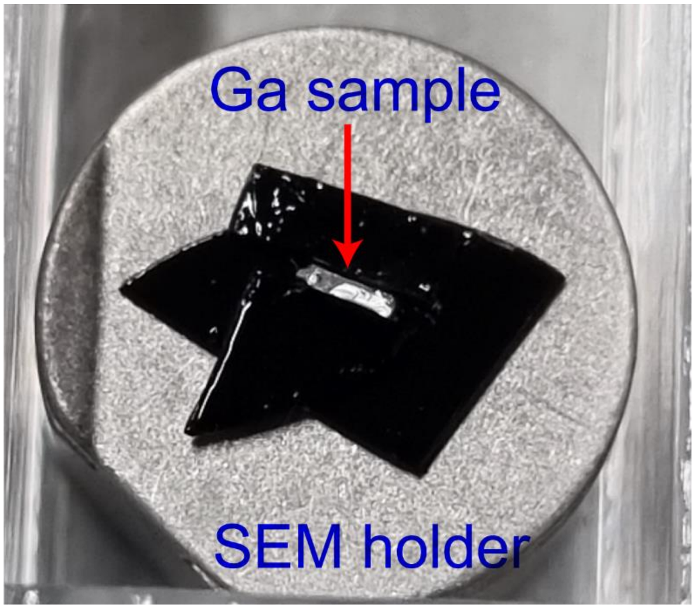

Figure S29. Photo of the SEM sample holder used in the cooling experiments. The sample holder enabled low temperature cooling of the specimen *in situ* to prevent any structural damage.
